# Investigation on the influence of the skin tone on hyperspectral imaging for free flap surgery

**DOI:** 10.1038/s41598-024-64549-9

**Published:** 2024-06-17

**Authors:** Ester Pachyn, Maximilian Aumiller, Christian Freymüller, Matthäus Linek, Veronika Volgger, Alexander Buchner, Adrian Rühm, Ronald Sroka

**Affiliations:** 1 Department of Urology, Laser-Forschungslabor, LIFE Center, University Hospital, LMU Munich, Fraunhoferstrasse 20, 82152 Planegg, Germany; 2 Department of Urology, University Hospital, LMU Munich, 81377 Munich, Germany; 3 Department of Otorhinolaryngology, University Hospital, LMU Munich, 81377 Munich, Germany

**Keywords:** Hyperspectral imaging, Perfusion, Melanin, Skin pigmentation, Skin tone, Free flap, Medical research, Optics and photonics

## Abstract

Hyperspectral imaging (HSI) is a new emerging modality useful for the noncontact assessment of free flap perfusion. This measurement technique relies on the optical properties within the tissue. Since the optical properties of hemoglobin (Hb) and melanin overlap, the results of the perfusion assessment and other tissue-specific parameters are likely to be distorted by the melanin, especially at higher melanin concentrations. Many spectroscopic devices have been shown to struggle with a melanin related bias, which results in a clinical need to improve non-invasive perfusion assessment, especially for a more pigmented population. This study investigated the influence of skin tones on tissue indices measurements using HSI. In addition, other factors that might affect HSI, such as age, body mass index (BMI), sex or smoking habits, were also considered. Therefore, a prospective feasibility study was conducted, including 101 volunteers from whom tissue indices measurements were performed on 16 different body sites. Skin tone classification was performed using the Fitzpatrick skin type classification questionnaire, and the individual typology angle (ITA) acquired from the RGB images was calculated simultaneously with the measurements. Tissue indices provided by the used HSI-device were correlated to the possible influencing factors. The results show that a dark skin tone and, therefore, higher levels of pigmentation influence the HSI-derived tissue indices. In addition, possible physiological factors influencing the HSI-measurements were found. In conclusion, the HSI-based tissue indices can be used for perfusion assessment for people with lighter skin tone levels but show limitations in people with darker skin tones. Furthermore, it could be used for a more individual perfusion assessment if different physiological influencing factors are respected.

## Introduction

Flap surgery is a profound part of head and neck cancer treatment^1,2^. Since the most common reason for flap loss is thrombosis^1^, it is important to properly monitor the perfusion of local and free flaps since tissue hypoxia can lead to tissue damage and necrosis^3–5^. Early detection of vascular problems with a monitoring device can help to intervene in time and save the transplant^3–5^. The current gold standard for tissue perfusion assessment is the clinical evaluation of the skin color, capillary refill time, tissue turgor and temperature of the flap and performing Doppler sonography^6^, but observed changes considering flap color and capillary refill can be overshadowed by high levels of skin pigmentation^7^. Since free flap transplant failures tend to happen mostly within 48 h after surgery and become rare as soon as the first post-operative week has passed^1^, a non-contact real-time imaging device, which can indicate perfusion correctly in the course of the post-operative time without being influenced by melanin would be most advantageous.

There are standard optical techniques in clinical use to determine tissue perfusion. Tissue oximeters working with near-infrared spectroscopy (NIRS) are used to detect complications in flap perfusion by measuring the tissue oxygen saturation (StO_2_). Near-infrared light (700–1000 nm) is sent onto the tissue to be absorbed, respectively, by hemoglobin (Hb) and melanin^8–10^, and scattered within the tissue. Unlike pulse oximeters, which only provide information on arterial oxygen saturation (SpO_2_)^11,12^, NIRS obtains perfusion information from additional chromophores^10,13^. Unfortunately, this method has a restricted spatial scanning resolution (1 mm), making it very time-consuming to image a complete surface via scanning with the probe attached to the skin^13,14^. Furthermore, additional imaging modalities that provide anatomical information are needed for the correct interpretation and analysis of the NIRS measurement, which increases the complexity of this method.

Regarding pigmentation, it has been stated that the Hb-signal derived from NIR light can be diminished by melanin^10^. A current study points out a linear correlation between NIR-fluorescence and low in vitro melanin concentration and that NIR fluorescence increases with skin phototype^15^, emphasizing that the amount of melanin must be considered for these measurement techniques.

Remote photoplethysmography (rPPG) also provides information on tissue perfusion by time-dependent imaging of remitted light with a contactless optical sensor^16–20^. The intensity of the remitted light depends on the number of red blood cells and the pulsatile movement of arterial and venous blood within the imaged tissue area^17^. The remitted light is usually recorded by a camera as a sequence of RGB images^16,17,19^. The time-dependent rPPG signals are calculated from the resulting RGB images, and spatially resolved perfusion signal results can be directly visualized intraoperatively during surgery^16,17,19,20^. This method can depict the development of free flap perfusion over time and may be used to create blood flow maps^18^.

However, it has been stated that the utilized visible light signals might not sufficiently penetrate into the regime of the pulsatile arterioles for every individual. Thus, the information obtainable about the real vascularization is limited, especially when considering the varying information reported in literature regarding the maximal penetration depth for red light ranging from 1.8 to 2.5 mm^19^. Furthermore, the rPPG method is sensitive to motion and illumination artefacts on the RGB images, which may lead to miscalculation of the rPPG signal changes.

Another approach for perfusion assessment includes laser Doppler flowmetry, laser Doppler imaging and speckle imaging^21–24^. All three methods are based on the Doppler effect, which describes a change in the wavelength of light proportional to the speed of the observed object, e.g. red blood cells^21,22^. This means that information on blood flow is provided but not on oxygen saturation or other chromophores. The influence of pigmentation or melanin content on the overlaying skin has not been described so far.

Perfusion measurement was also done using infrared thermography, which is usually performed to measure skin temperature. Since skin temperature correlates with the perfusion of the flap, it can be used to estimate the blood flow within the tissue^7^. However, the sensitivity of the measurement results of this method is highly influenced by the amount of subcutaneous fat at the measured body site^25^.

In comparison to the previously mentioned methods, hyperspectral imaging (HSI) allows perfusion measurement within a few seconds, without skin contact and over a larger area^6,26^. A spatially resolved image with the individual measured spectral information for each pixel in the image can be recorded^27^. This provides more detailed information on the tissue properties and early changes in its optical characteristics^27^. Depending on the device, HSI records up to hundreds of narrow spectral bands ranging from ultraviolet (UV) to NIR-light, which highly increases the detected signal information compared to multi-spectral applications^27–29^. HSI has already been shown to be a reliable method for controlling blood perfusion in free flaps^6,30,31^.

**Figure 1:**
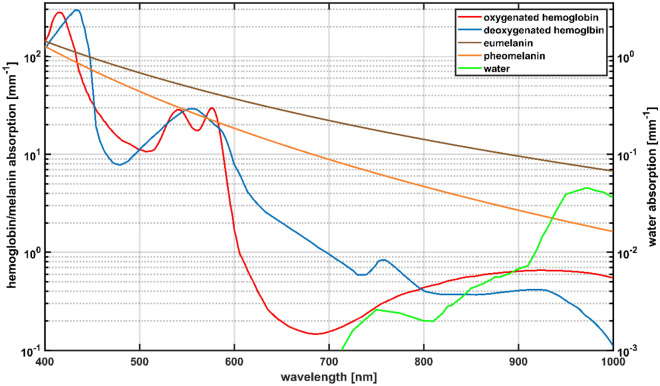
The absorption spectra of melanin, the hemoglobin (Hb) species and water, adapted from^33,34,36,37^.

HSI uses the principle of diffuse remission spectroscopy, which means it is also dependent on the optical properties of the tissue and especially on the content of the different tissue chromophores^32^. When looking at the absorption spectra of the most profound chromophores (Fig. 1)^33,34^, oxygenized Hb (Oxy-Hb) has characteristic absorption maxima at 415 nm, 542 nm and 577 nm^35^. In contrast, deoxygenized Hb (Deoxy-Hb) has its absorption maxima at 430 nm, 555 nm and 756 nm^9,35^.

There are two types of melanin that determine human skin pigmentation, eumelanin and pheomelanin, with slightly different absorption spectra (Fig. 1)^33,34,36,37^. Even though it has been assumed for a long time that the ratio of eu- to pheomelanin is highly on the side of eumelanin with around 90%^38,39^, recent studies show that the ratio of the melanin types is around 26% for pheomelanin and around 74% for eumelanin^39–41^. It is claimed otherwise that the amount of melanin highly varies among different individuals, even when coming from the same regional background^34^. The absolute amount of melanin is dependent on the ethnic origin and the resulting individual genetic setup as well as on the exposure to UV light^39,42–44^. The absorption coefficient of both melanin types is higher for almost all wavelengths compared to the absorption spectrum of Oxy-Hb and Deoxy-Hb (Fig. 1)^33,34,36,37,45^. The overlap of the absorption coefficient of melanin and Hb has been reported to be the reason for misestimating a high melanin concentration as a high amount of Hb, possibly leading to wrong perfusion assessment^45^.

Due to the optical properties of melanin, wrong estimations of the perfusion of flaps with HSI in a more pigmented population cannot be excluded. This aims in a clinical request to provide objective and valid perfusion assessment in free flaps independent of pigmentation. Therefore, the present investigation will focus on the question of whether skin pigmentation has an influence on tissue indices determined using a medically approved hyperspectral imaging system.

In order to find variations caused by different skin properties of different body sites, a total of 16 body sites of varying skin structure were measured by HSI. Since one of the commonly used flaps for oro-maxillofacial reconstruction is the radial and ulnar forearm flap^30,46–48^, this study is going to focus on the results of the measurements of the volar forearm. To highlight the influence of melanin on the measurements, the forearm results will be compared to those of a less pigmented body side. Furthermore, other factors that might be responsible for measurement variation, such as age, sex, BMI, and smoking, will be evaluated.

## Material and methods

In this work, an observational diagnostic clinical study was performed. The investigation complies with the principles of the Declaration of Helsinki and was approved by the Ethical Review Board of the Ludwig-Maximilians-University, Munich, Germany (no. 21-0865). Every participant was of legal age, and informed consent was signed before participation.

### Hyperspectral imaging device

Data acquisition was performed using an HSI-device (TIVITA^®^ Tissue, Diaspective Vision GmbH, Am Salzhaff/Pepelow, Germany) that emits light from six halogen lamps^49^ and detects remitted light in the wavelength range of 500–1000 nm^9,26^. The dispersive element within the device splits the remitted light into 100 spectral bands of 5 nm increments^9,26^. This system generates spectral images using a modified^9^ pushbroom method^27^. The spectral and spatial information are acquired by scanning via a slit along the first coordinate axis, with the second axis being fully mapped for each slit position^21,22,27^. Each image created by the HSI-device has a size of 480 × 640 pixels, forming the three-dimensional datacube with the size 480 × 640 × 100 (X × Y × wavelength)^26^. The HSI-device is calibrated in the factory by the manufacturer and does not need further calibration before usage, as the calibration data is stored within the system.

**Figure 2:**
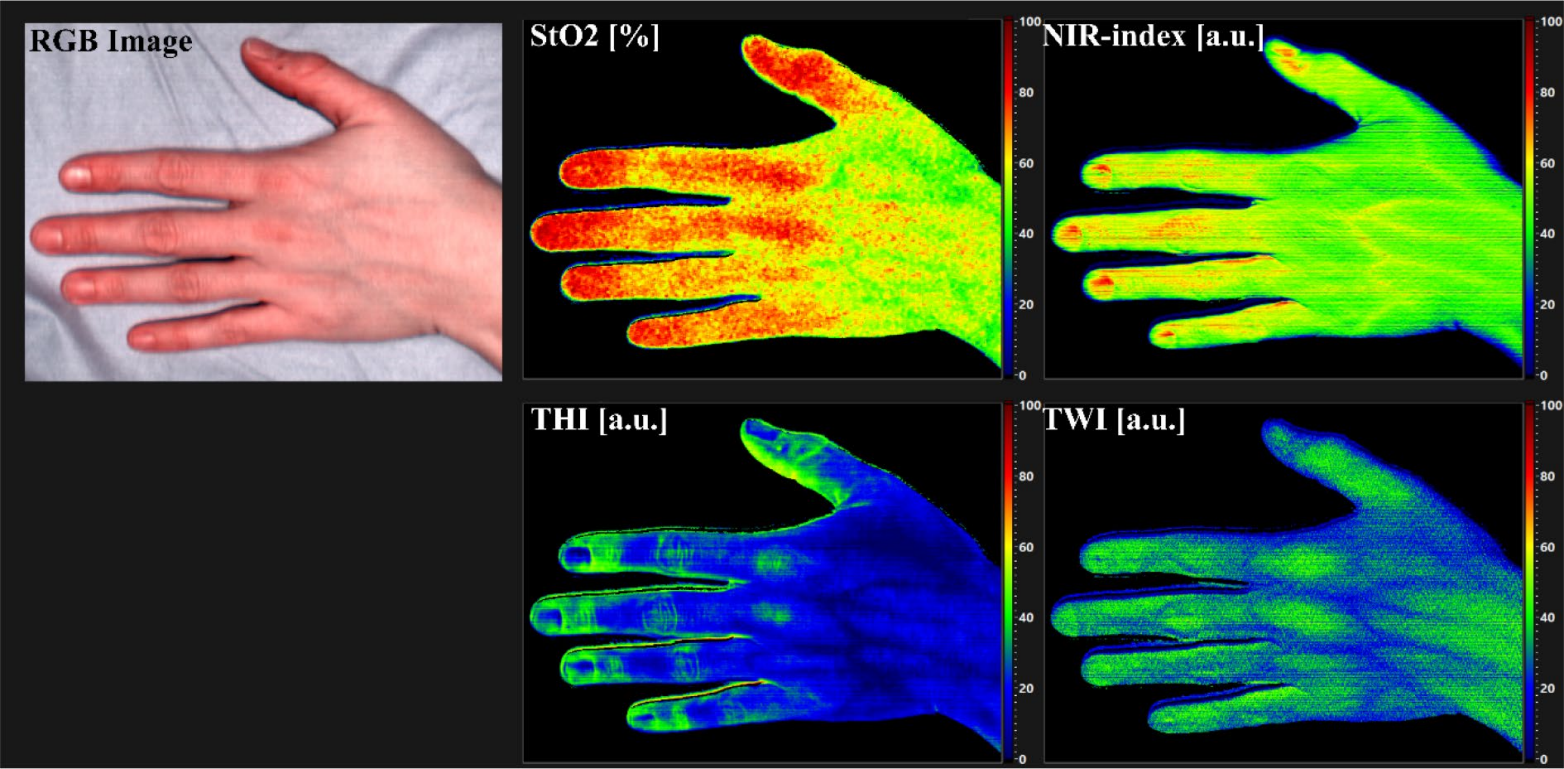
Results for the false color images of the four tissues indices StO_2_, NIR-index, THI and TWI with the RGB image as shown by the HSI-device.

As the main evaluation procedure, the system's internal software (TIVITA^®^ Suite 1.5, Diaspective Vision GmbH, Am Salzhaff/Pepelow, Germany) analyses the spectral data to derive four tissue-specific indices^49^. These include tissue oxygen saturation (StO_2_) given in percent, the Near-Infrared-Perfusion-Index (NIR-index), which describes perfusion in the deeper layers of the tissue, the Tissue-Water-Index (TWI), and the Tissue-Hemoglobin-Index (THI) all given in arbitrary units (a.u.) ranging from 0 to 100 where 0 is equivalent to low parameter content and 100 to high parameter content^26,49^. Figure 2 shows the result of an HSI measurement, the RGB image and the four false color images of the indices as the system presents them.

The light penetration depth of tissue regarding different wavelengths ranges from 0.8 mm at λ = 500 nm to 2.6 mm at λ = 1000 nm^9^. The measured remission spectra are converted into absorbance spectra $A$ by the HSI-software for the calculation of the tissue indices. To calculate the NIR-index, the mean absorbance of two wavelength ranges in the near-infrared at λ = (655–735) nm and at λ = (825–925) nm are put in a ratio (Eq. 1)^9^. As one of the characteristic absorbance maxima of water is located at λ = 970 nm, the TWI is calculated as shown in Eq. (2) by putting the mean absorbance in the wavelength range of λ = (880–900) nm in relation to that in the range of λ = (955–980) nm^9,50,51^. The THI is generated similarly using the mean absorbance in the wavelength ranges from λ = (530–590) nm and λ = (785–825) nm (Eq. 3)^9^.

The StO_2_ value is calculated by comparing the minima of the second derivation of the calculated absorbance at characteristic wavelength ranges of hemoglobin, see Eq. (4)^9^. Since light in this wavelength range does not penetrate deep into the tissue, only superficial oxygen saturation can be derived.

1 $$NIR-index= \frac{\frac{mean (A \left[825 nm\dots 925 nm\right])}{mean(A [655 nm\dots 735 nm])}-{s}_{1}}{{s}_{2}-{s}_{1}}$$

2 $$TWI= \frac{\frac{mean (A \left[955 nm\dots 980 nm\right])}{mean(A [880 nm\dots 900 nm])}-{s}_{1}}{{s}_{2}-{s}_{1}}$$

3 $$THI= \frac{\frac{mean (A \left[785 nm\dots 825 nm\right])}{mean(A [530 nm\dots 590 nm])}-{s}_{1}}{{s}_{2}-{s}_{1}}$$

4 $$StO2= \frac{\text{min}\left({A}^{{\prime}{\prime}}\left[570 nm\dots 590 nm\right]\right)/{r}_{1}}{\text{min}\left({A}^{{\prime}{\prime}}\left[570 nm\dots 590 nm\right]\right)/{r}_{1}+\text{min}\left({A}^{{\prime}{\prime}}\left[740 nm\dots 780 nm\right]\right)/{r}_{2}}$$

*s*_1_, *s*_2_ in Eqs. (1–3) and *r*_1_, *r*_2_ in Eq. (4) are additional scaling and calibration factors that are under disclosure by the developers. In the end, the tissue indices are presented as spatially resolved false color images simultaneously with an RGB image^49^. The displayed RGB image serves as a spatial reference image for the false color images and is automatically generated by the system's internal software using an external calibration by the manufacturer included in the system. It should be noted that these tissue indices do not describe the absolute amount of, e.g. water or Hb, but can be used as a reference to detect a change in perfusion when reassessing the perfusion of the tissue^9,26,49^. The acquisition of one datacube, including the display of an RGB and false color images for the calculated tissue indices, takes about 6 s^26^.

### Skin type classification

Skin type classification was performed by means of the Fitzpatrick Skin Type questionnaire for pigmentation classification using the version contributed by Prof. Bhupendra C. K. Patel MD, FRCS^52^. The classification into Fitzpatrick classes (F-Classes) is based on a scoring system regarding skin and hair color as well as burning and tanning tendencies^52^. Since all people classified as F-Class V or VI scored > 32 points in the questionnaire, these participants were grouped in the current study.

**Figure 3:**
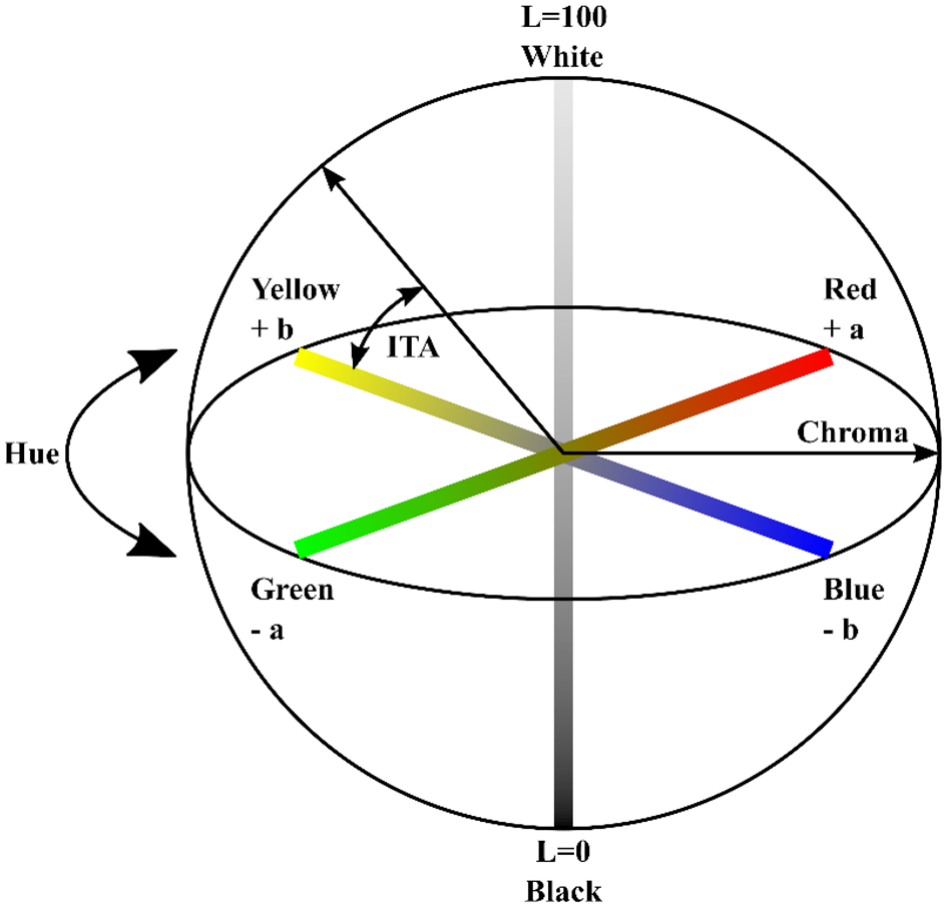
The CIELAB color space with L = luminance, a* = red and green values, b* = yellow and blue values, hue = angle on the chromaticity axes, chroma = color saturation, adapted from^55^.

In order to assess the degree of pigmentation quantitatively, the individual typology angle (ITA) (Table 1)^53^ was determined from the HSI-device generated RGB images. The ITA is based on the CIELAB color space system, defined by the Commission International d’Eclairage in 1976^54^. This three-dimensional color space (Fig. 3)^55^ is supposed to describe differences in luminance (L*) independently from color (a* and b*) and is not device-dependent^54,56^. Herein the factors a* and b* relate to Hering’s opponent color theory, and in this regard, the a*-axis describes values from the red to the green range, whereas the b*-axis describes values from the blue to the yellow range^54,56^. The ITA is than calculated using Eq. (5)^53^:

**Table 1:**
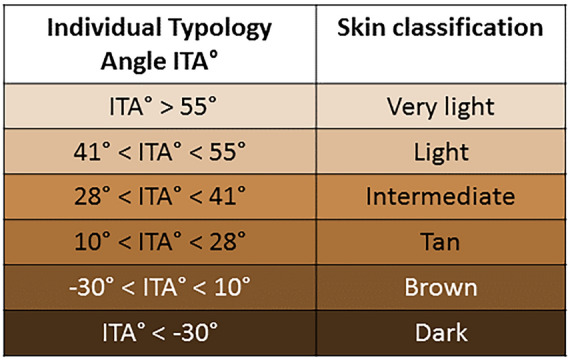
The ITA regarding skin pigmentation classification, adapted from^53^.

5 $$ITA^\circ = [arctan({L}^{*} - 50)/{b}^{*}] \times 180/\uppi$$

It has to be noted that the ITA values do not correspond directly to the six F-Classes^57,58^.

### Subjects

The collected data was derived from 101 participants with a distribution as even as possible of age (median = 32a mean = 38.5a, range: 18a–83a), sex (male n = 51, female n = 50), BMI (median = 24.8 kg/m^2^, mean = 25.5 kg/m^2^, range 16.8–37.9 kg/m^2^) and smoking habits (47 current or former smokers vs. 53 non-smokers). To get valuable results, people with known blood diseases such as acute or chronic anemia (Hb value < 10 g/dl), polyglobulia (Hb value > 16 g/dl for females, > 17 g/dl for males), and porphyria were excluded as well as people who had to undergo current or recent radiation therapy or long-term treatment with topical steroids. In total, nine people were classified as F-Class I, n = 25 as F-Class II, n = 27 as F-Class III, n = 24 as F-Class IV and n = 16 as F-Class V/VI. A total of 16 body sites were measured: left and right dorsi of the hands, palms, volar forearms, dorsum of the feet, soles, heels and lumbar back regions and on point at the center of the abdomen and side of the neck.

### Study execution

**Figure 4:**
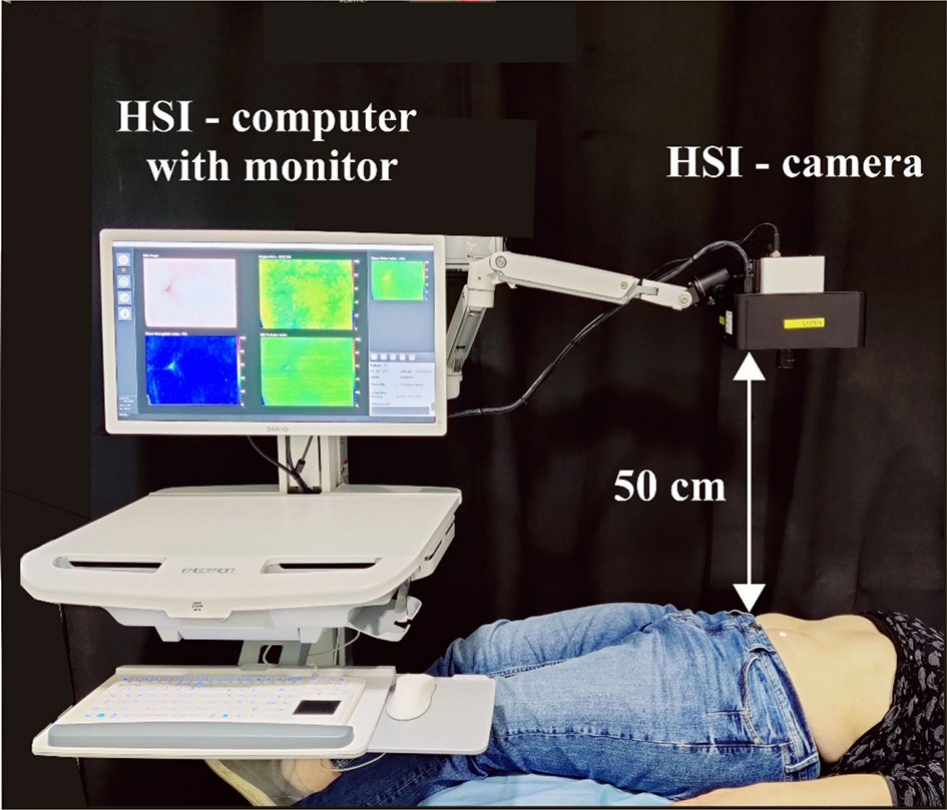
Setup for a measurement taken with the HSI-device.

Data collection was conducted at the Department of Otorhinolaryngology, University Hospital, LMU Munich, from November 2021 to Mai 2022. Each participant was questioned on their age, sex, weight, height, if they were smoking, last time of tanning (more than 3 months ago was considered untanned^52^), exclusion criteria and if there were any other relevant medical characteristics for the measurements. The Fitzpatrick classification questionnaire^52^ was used for standard skin classification. HSI-measurements were performed in a darkened room, where the patients lay flat on an examination table and had 5 min of rest before contemplating the measurements. In Fig. 4, the experimental setup is shown. The HSI-camera is positioned in 50 cm distance from the tissue surface. Therefore two guidance LEDs included in the camera head are used. Shadows or other artefacts can distort the data acquisition. To avoid shadows caused by natural body curves, the regions chosen for the measurements were as even and free from shadows as anatomically possible. Any recordings with visible movement or other artefacts were replaced with suitable ones within the session. Altogether, one complete data acquisition session took between 30 and 45 min.

### Data acquisition

The false color images generated with the HSI-device were transferred into MATLAB (version 2018b The MathWorks Inc., Natick, Massachusetts, USA) to derive the NIR-index, StO_2_, THI and TWI tissue indices. For each body part, a square region of interest (ROI) of 50 × 50 pixels, corresponding to 15 × 15 mm^2^ on the skin surface, was selected based on a standardized method adapted from a recently published study^59^. The ROI was defined near the center of the image, and no visible blood vessels or shaded areas at the edge of the skin surface were included in the analyzed area, as this could influence the results for the calculation of the tissue indices and reduce the comparability of the results. The mean and standard deviation for all tissue indices and the RGB values were calculated for the area within the ROI. Over all measurements, the median standard deviation for the obtained tissue indices was 6% for the NIR-index, 9% for StO_2_, 17% for THI and 9% for TWI. Based on the RGB values of the ROI, the ITA was derived. Therefore, the corresponding LAB color values were calculated using the mean RGB. This conversion from RGB to LAB was done by a custom-written program using the MATLAB internal function rgb2lab with options WhitePoint d50 and ColorSpace srgb (Supplement 8). Using Eq. (5), the ITA was derived from the LAB values. The ITA was assigned for each evaluated body site individually.

### Statistical methods

The ITA, the F-Classes, and the possible influencing factors were statistically analyzed concerning the four tissue indices with the statistic software SPSS (IBM^®^ SPSS^®^ Statistics Version 27, IBM, Deutschland GmbH, Ehningen, Germany). A two-sided H-Test with Bonferroni correction for multiple testing was performed to figure out statistically significant differences between the tissue indices of the participants after sorting them according to the five F-Classes. In addition, the mean values and the standard deviation of the tissue indices sorted according to the F-Classes were calculated for the different body sites. To investigate coherency between the ITA, age or BMI and the tissue indices, a two-sided Spearman’s Rank correlation test with correlation coefficient r was performed. The Mann–Whitney-U-Test was applied to see differences in the measurement results considering the sex or the smoking behavior of the participant. The influence of the last tanning time was not further investigated since the study occurred in the second winter of the COVID-19 pandemic; therefore, not enough participants had relevant recent sun exposure. For all tests, a p-value < 0.05 was considered significant.

### Institutional review board statement

The study was conducted according to the guidelines of the Declaration of Helsinki and approved by the Ethics Committee of Ludwig-Maximilians-University, Munich, Germany (no. 21-0865).

### Informed consent and consent for publication

Informed consent was obtained from all participants involved in the study. Consent for publication was obtained from all participants within the informed consent.

## Results

### Measurements

The measurements were tolerated well by the 101 participants despite a few exceptions. One measurement for the left and right lower back were not acquired for one volunteer of F-Class V/VI as the participant did not allow to be photographed there. One participant from F-Class V/VI did not allow any measurements on the feet. One measurement of the right sole of the foot in F-Class IV and one measurement of the right dorsum of the foot in F-Class III could not be conducted appropriately. Overall, this results in 99 participants for the dorsum and the sole of the right foot and in 100 participants for the lumbar back regions, the sole and dorsum of the left foot and both heels.

### Comparison of Fitzpatrick class and ITA to the tissue indices

**Figure 5:**
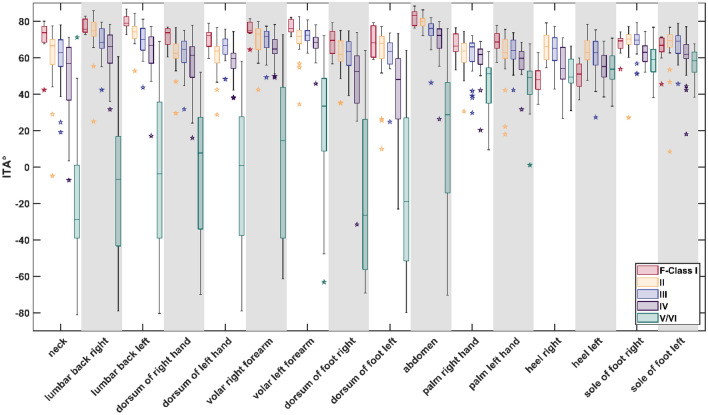
Variation of the ITA in relation to the individual Fitzpatrick Classes (F-Classes).

Comparison of the evaluated F-Class of every participant to the ITA shows for the single F-Classes that the ITA angle differs most for the F-Class V/VI between the different body sites, especially when comparing physiologically pigmented to unpigmented body side (unpigmented body sites: palms of the hands, soles of the feet, heels). For pigmented sites, F-Class V/VI ranges from ITA 60 to − 80, for unpigmented from 80 to 30 (Fig. 5). Similar trend can be seen for F-Class IV. On the unpigmented body sites, the overall ITA range is 80–30, while at pigmented a range from 88 to − 80 is observed. The comparison between ITA and all body sites can be found in the supplementary material (Supplement 1, Supplementary Fig. S1). Overall, for almost all sides and comparisons a significant difference was observed when comparing the ITA values of F-Class V/VI and IV to those of other classes. Exception where both heels, and the soles of both feet, here F-Class V/VI did not have any significant difference to I and IV had only partial a significant difference to lower classes. Between the F-Classes III II and I no significant difference was found for all sites, except between F-Class I and II at both heels. (See Supplement 2 for all results).

In the following sections, the HSI-derived tissue indices are shown compared to F-Class and ITA for the volar left forearm as example for a pigmented body, also the most common site for skin flap extraction, and for the sole of the right foot as unpigmented site.

#### The tissue indices compared to Fitzpatrick Classes

**Figure 6:**
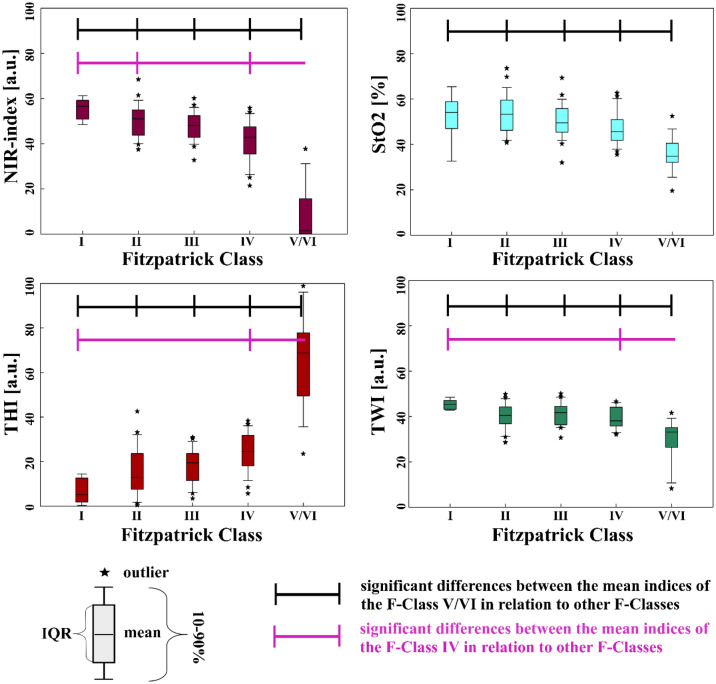
Tissue indices distribution regarding the Fitzpatrick Classes, volar left forearm.

The values of all four tissue indices were not evenly spread over the F-Classes. This difference was apparent for all generally more pigmented body parts (the volar forearms left and right, the dorsum of the hands and feet left and right, the lumbar back region left and right, the abdomen and the neck). Yet, the difference between a tissue index values was only significant (p < 0.05) between some F-Classes. In Fig. 6, the distribution of the tissue indices over the F-Classes calculated for the volar left forearm is shown.

The most striking tendency is that the tissue indices of the higher-numbered F-Classes differ significantly from those of the lower-numbered F-Classes, especially F-Class V/VI. E.g., at the left forearm, the NIR-index, StO_2_ and TWI decrease, with the F-Class number having the lowest values at F-Class V/VI. The THI values show an opposite, increasing trend with the highest values for F-Class V/VI. This trend is visible for all more pigmented body parts.

In contrast, hardly any significant differences can be observed between the values of the tissue indices of the directly following lower-numbered F-Classes. All significant differences between the tissue indices of the different F-Classes at the body sites are listed in the supplementary content (Supplement 3). An overview of the mean tissue index values sorted into F-Classes I–V/VI for all pigmented body sites under investigation can be found in the supplementary content (Supplement 4).

**Figure 7:**
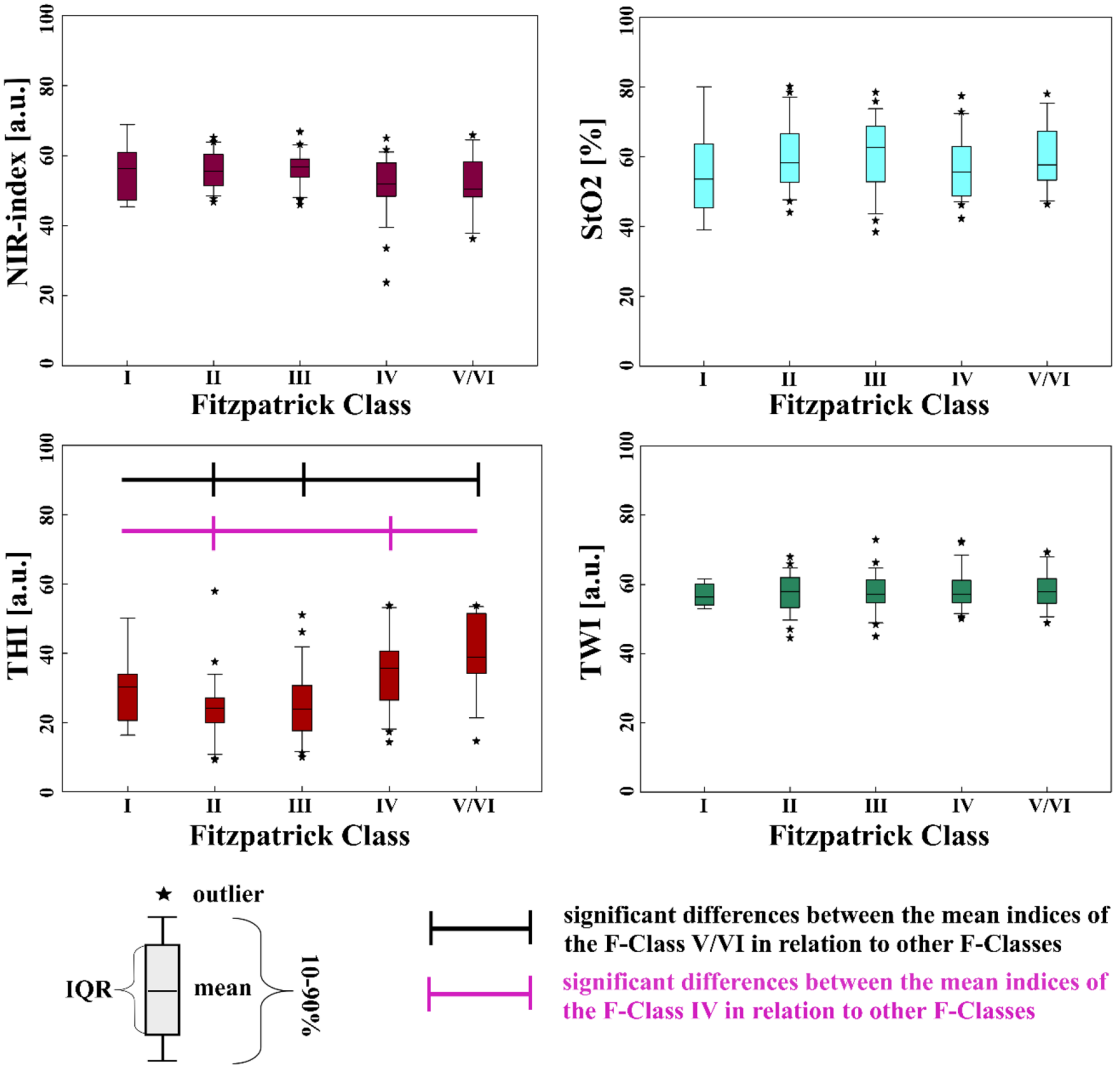
Tissue indices regarding the F-Classes, sole of the right foot.

In contrast to pigmented body sites, for the generally less pigmented ones (palms, heels and soles of the feet), the amount of significant differences between the tissue index values sorted according to their F-Classification is not as striking as for pigmented body parts. When looking, e.g. at the sole of the right foot (Fig. 7), only the THI values differ significantly between F-Class V/VI and III, F-Class V/VI and II and between F-Class IV and II. When comparing the NIR-index, StO_2_ and TWI, the values do not show significant differences among the F-Classes.

An overview of all significant differences between tissue indices for the F-Classes for less pigmented sites is shown in Supplement 5. The mean tissue index values for the 6 generally less pigmented body sites are shown in Supplement 6.

#### The tissue indices compared to ITA

**Figure 8:**
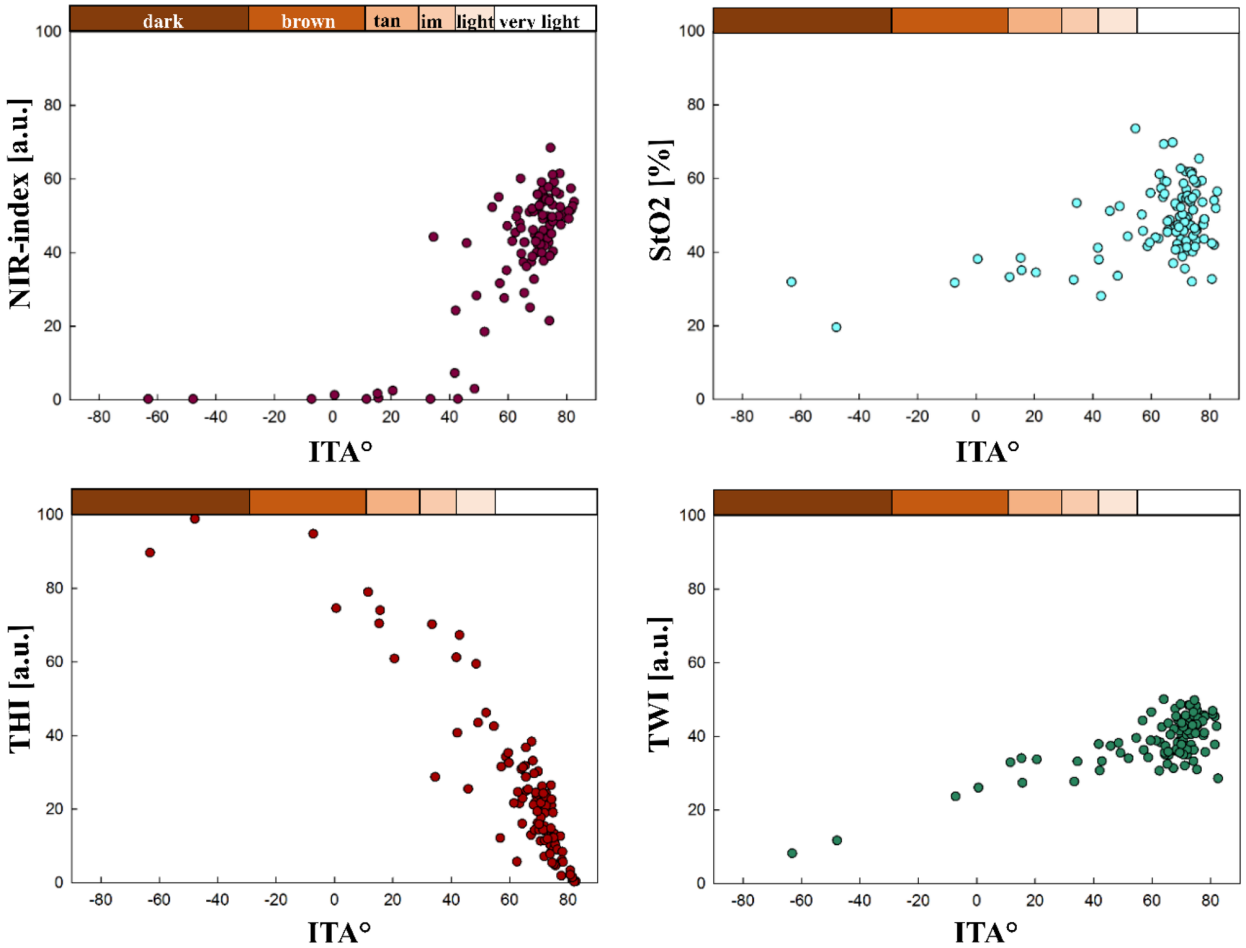
Tissue indices distribution regarding ITA° and ITA skin classification (upper x-axis), volar forearm left; (im: intermediate).

The correlation of the tissue indices to the ITA are significant, e.g., for the left volar forearm, where ITA shows a correlation to NIR-index (r = 0.626), StO_2_ (r = 0.283), THI (r = − 0.839) and TWI (r = 0.525). The relations between the ITA and the tissue indices for the volar left forearm are shown in Fig. 8.

**Figure 9:**
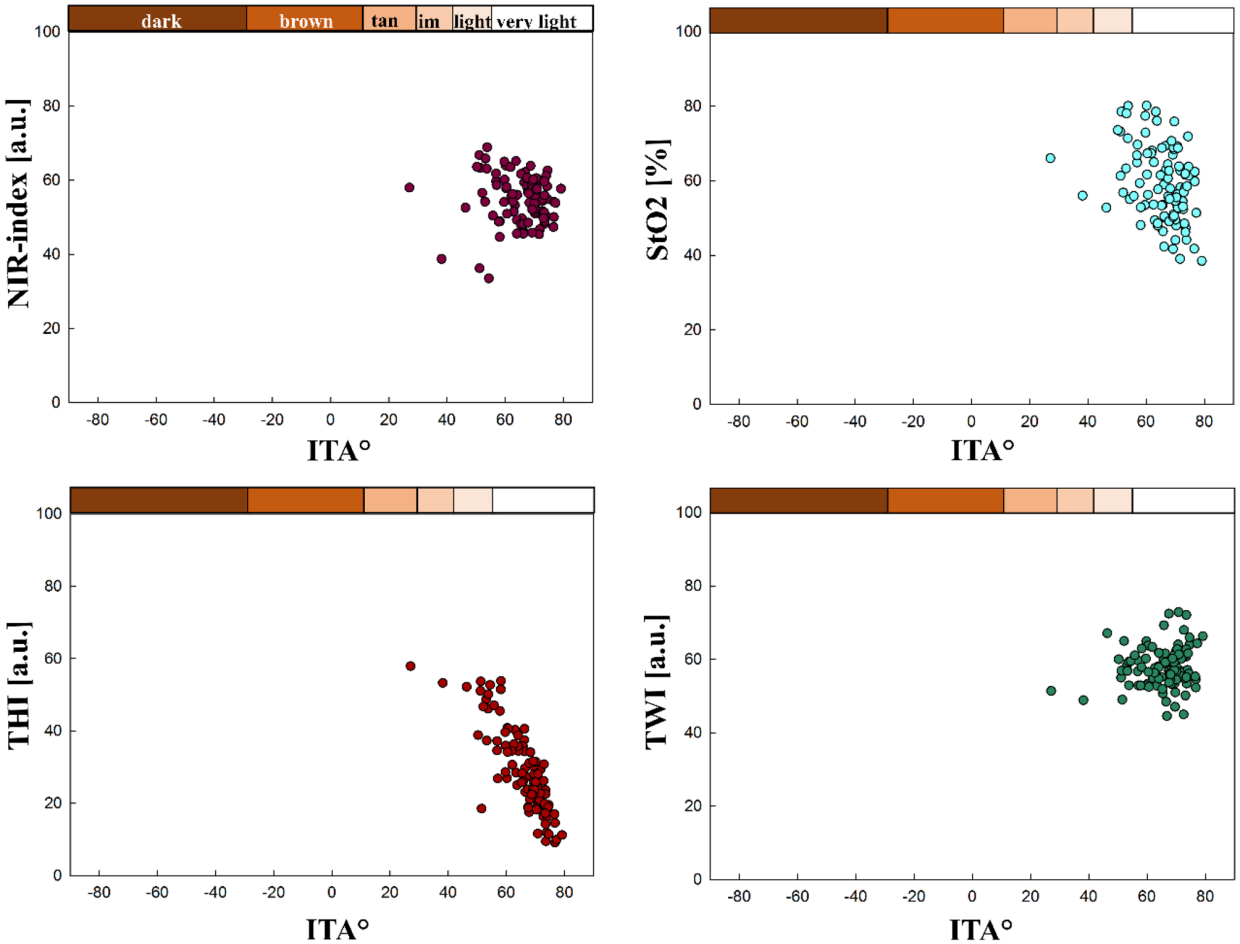
Tissue indices distribution regarding ITA° and ITA skin classification (upper x-axis), sole of the right foot.

In contrast, for the sole of the right foot, there is only a significant correlation between ITA and StO_2_ (r = − 0.344) and between ITA and THI (r = − 0.830). The relations between the ITA values and the tissue index values are exemplary shown for the sole of the right foot in Fig. 9. For the sole of the left foot, only a significant correlation between ITA and THI (r = − 0.866) could be observed. For the palms, a significant correlation between ITA and NIR-index (r = 0.364 left palm, r = 0.358 right palm), as well as ITA and THI (r = − 0.838 left palm, r = − 0.801 right palm), could be found. For the heels, a significant correlation between ITA and NIR-index (r = − 0.347 left heel, r = − 0,387 right heel), ITA and StO_2_ (r = − 0.416 left heel, r = − 0.611 right heel) as well as ITA and THI (r = − 0.818 left heel, r = − 0.886 right heel).

Overall, the correlation between ITA and THI is strong for all body parts. The correlation between ITA and NIR-index is strong for all generally more pigmented body parts, moderate for the heels and palms and insignificant for the soles of the feet. For TWI and StO_2_, in general, the more pigmented body sites show strong to moderate correlations with ITA. The less pigmented sites show no or only weak correlation with these two tissue indices. When comparing the four tissue parameters with the ITA across all 16 body sites, no significant correlation was found in only 10 comparisons (10/160), all for non-pigmented body sites. An overview presenting all correlation results between ITA and the tissue indices for all body sites is given in Supplement 7.

### Investigations of further possible influencing factors on tissue indices

Next to melanin also age, BMI, sex, or smoking could possibly influence the tissue indices. But, as melanin appears to have a considerably high impact on the HSI-derived tissue indices, the effect of other influencing factors could be masked by the melanin. Therefore, the other influencing factors were investigated by correlating only the tissue index values of body parts with an ITA > 55°, defined as the lightest skin hue^53^.

When analyzing the influence of age on the tissue indices, it can be seen that age and THI values correlate negatively at both heels and soles of the feet (e.g. r = − 0.455, heel left). On contrary, a positive correlation becomes apparent with THI and the other body sites (e.g. r = 0.258 volar forearm left) except for the abdomen and the dorsum of the feet, where no significant correlation is observed. A negative correlation between age and ITA can be seen for these same body sites, except for the sole of the right foot, where no correlation is seen. Additionally, age positively correlates with TWI at the neck, palms, lumbar back regions, and the sole of the left foot (e.g. r = 0.311 lumbar back left). NIR-index correlates negatively at the left forearm (r = − 0.245), and StO_2_ correlates negatively at the heels, dorsum of the right foot, and dorsum of the left hand (e.g. r = − 0.418 back of the left hand). An overview of all the significant correlations is provided in Table 2

**Table 2:** Significant correlations between age and tissue indices at different body sites for skin areas with ITA > 55°.

Indices	Body site	n	r-value	p-value
ITA	Heel left	65	0.405	< 0.001
	Heel right	62	0.339	0.007
	Sole of foot left	83	0.274	0.012
	Dorsum of hand left	70	− 0.363	0.002
	Dorsum of hand right	66	− 0.367	0.002
	Forearm left	83	− 0.23	0.036
	Forearm right	82	− 0.408	< 0.001
	Palm left	70	− 0.409	< 0.001
	Palm right	71	− 0.367	0.002
	Neck	62	− 0.384	0.002
	Lumbar back left	78	− 0.355	0.001
	Lumbar back right	78	− 0.309	0.006
NIR-index	Forearm left	83	− 0.245	0.025
StO_2_	Heel left	65	− 0.319	0.01
	Heel right	62	− 0.283	0.026
	Dorsum of foot right	61	− 0.392	0.002
	Dorsum of hand left	70	− 0.418	< 0.001
THI	Heel left	65	− 0.455	< 0.001
	Heel right	62	− 0.318	0.012
	Sole left	83	− 0.326	0.003
	Sole right	86	− 0.215	0.047
	Dorsum of hand left	70	0.335	0.005
	Dorsum of hand right	66	0.34	0.005
	Forearm left	83	0.258	0.018
	Forearm right	82	0.36	< 0.001
	Palm left	70	0.297	0.013
	Palm right	71	0.31	0.009
	Neck	62	0.377	0.003
	Lumbar back left	78	0.401	< 0.001
	Lumbar back right	78	0.269	0.017
TWI	Sole left	83	0.233	0.034
	Palm left	70	0.356	0.002
	Palm right	71	0.309	0.009
	Neck	62	0.262	0.039
	Lumbar back left	78	0.311	0.006
	Lumbar back right	78	0.243	0.032

Regarding BMI, observations similar to the ones above can be made when analyzing THI (e.g. r = 0.534 left palm) and ITA (e.g. r = − 0.528 left palm) measurements at the palms. At the neck, BMI and THI correlate positively.

BMI correlates positively with the NIR-index at the soles of the feet (e.g. r = 0.227 right sole) but negatively at the neck (r = − 0.366). BMI correlates positively with StO_2_ at the soles of the feet (e.g. r = 0.245 sole of the right foot) but negatively at the dorsum of the right hand. At the lumbar back regions, BMI also correlates positively with TWI but negatively with the dorsum of the right hand (e.g., r = 0.337 at the right lumbar back region).

Significant correlations between all body sites and BMI are provided in Table 3

**Table 3:** Significant correlations between BMI, ITA and tissue indices at different body sites for skin areas with ITA > 55°.

Indices	Body site	n	r-value	p-value
ITA	Palm left	70	− 0.528	< 0.001
	Palm right	71	− 0.354	0.002
NIR-index	Sole left	83	0.255	0.02
	Sole right	86	0.227	0.035
	Neck	62	− 0.366	0.003
StO_2_	Sole left	83	0.295	0.007
	Sole right	86	0.245	0.023
	Dorsum of hand right	66	− 0.245	0.047
THI	Palm left	70	0.534	< 0.001
	Palm right	71	0.317	0.007
	Neck	62	0.398	0.001
TWI	Lumbar back left	78	0.332	0.003
	Lumbar back right	78	0.337	0.003
	Dorsum of hand right	66	− 0.248	0.044

Regarding sex, women have lower mean StO_2_ values than men at the forearms, lumbar back regions, abdomen, neck, right palm and dorsum of the hand. Women have mean StO_2_ values ranging between 40% at the abdomen to 46% at the volar left forearm and 64% at the right palm. Men had mean StO_2_ values ranging between 46% at the abdomen, 54% at the left forearm and 69% at the right palm. Depending on the body site, the saturation differences between the sexes vary from 5% at the right palm and back of the hand to 8% at the forearms and 11% at the lumbar back region and neck.

TWI values are slightly higher for women than men at the abdomen (48 for women vs. 46 for men) and the left palm (49 for women vs 47 for men). The mean index values of all significant body parts are listed in Table 4

**Table 4:** Significant correlations between sex and tissue indices at different body sites for skin areas with ITA > 55°.

Indices	Body site	N male	Mean tissue index male	N female	Mean tissue index female	p-value
NIR-index [a.u.]	Forearm left	39	49	44	46	0.042
	Forearm right	37	50	45	44	0.001
	Dorsum of hand right	30	60	41	54	< 0.001
	Palm left	29	57	41	54	0.017
	Palm right	30	58	41	52	< 0.001
	Heel right	34	57	28	60	0.032
StO_2_ [%]	Forearm left	39	54	44	46	< 0.001
	Forearm right	37	55	45	47	< 0.001
	Dorsum of hand right	30	60	41	54	0.033
	Palm right	30	69	41	64	0.033
	Neck	23	69	39	58	< 0.001
	Lumbar back left	37	56	41	45	< 0.001
	Lumbar back right	34	55	44	44	< 0.001
	Abdomen	38	46	46	40	0.001
	Heel right	34	63	28	70	0.009
TWI [a.u.]	Palm left	29	47	41	49	0.016
	Abdomen	38	46	46	48	0.03

No significant differences concerning the tissue indices could be found between smokers and non-smokers.

## Discussion

From the results of this study, it is apparent that HSI-derived tissue indices are not necessarily robust against skin tone influences. This is an important result to be considered in clinical examinations relying on HSI-derived tissue indices, in order to avoid wrong conclusions about the physiological state of a tissue region for patients with different skin tones. In detail, correlations between four HSI-derived tissue indices (NIR-index, StO_2_, THI, TWI) and two established skin tone classifiers (F-Class and ITA) were investigated for different body sites. The two skin tone classifiers contain some but very different information about the physiological state of a tissue region. F-Class is not body-site-specific and only relevant for the characterization of pigmented body sites. As expected, the ITA seems to be better suited than the F-Class to correct a skin-tone-related bias of tissue indices at a particular body site.

When analyzing the relationships between the HSI-derived tissue indices and ITA, BMI, age and sex, the ITA showed the largest number of significant relationships. This means that skin tone, and thus melanin, is the main influencing factor for the tissue indices calculated by the HSI-system. This and other possible influencing factors and their relationship with the tissue indices are discussed in more detail in “Melanin derived bias”. “Age derived bias”, “BMI derived bias”, “Sex derived bias”, “Smoking derived bias”. Subsequently, future perspectives and limitations will be discussed in "The importance of the findings for future research" and "Limiting factors".

### Melanin derived bias

#### Indications and explanations for a melanin influence on HSI tissue indices

The results for the tissue indices, especially compared to ITA, strongly indicate that melanin affects Hb-related measurements, as reported for other non-invasive measurement techniques^60–67^. The software potentially indicates falsely low NIR-index, StO_2_, as well as falsely high TWI values. This phenomenon can be explained by comparing the absorption spectra of the different melanin and hemoglobin species and water (see Fig. 1) within the wavelength ranges used to calculate the tissue indices shown in Eqs. (1–4).

The explanations are not impaired by the fact that the calibrations and scaling factors in these equations are not disclosed by the manufacturer.

According to our results for the NIR-index and TWI, both tissue indices decrease with decreasing ITA or for higher F-Class at generally more pigmented body sites. For the calculation of NIR-index Eq. (1) and TWI Eq. (2) the absorbance at the shorter wavelength is used in the denominator. The corresponding absorbance ratios of eumelanin are $\frac{{A}_{Eumelanin} [825 nm \dots 925 nm]}{{A}_{Eumelanin} [655 nm \dots 735 nm]} \sim 0.42$ for the NIR-index and $\frac{{A}_{Eumelanin} \left[955 nm \dots 980 nm\right]}{{A}_{Eumelanin} [880 nm \dots 900 nm]} \sim 0.75$ for the TWI, respectively (see Fig. 1)^34,36,37^. In case of low melanin content, the absorbance ratios of tissue (with its typical volume fraction of the main absorbing chromophores blood and water) are above 1^36^. Therefore, with increasing melanin concentration, the two absorbance ratios and so the NIR-index and TWI reduce compared to tissue with very low melanin content. As the absorbance ratio of eumelanin is even smaller for the NIR-index than for the TWI (0.42 vs. 0.75), the NIR-index is affected more strongly by the presence of eumelanin and even becomes 0 at low ITA.

Concerning the THI, increasing values were observed with decreasing ITA. For compared wavelength ranges of the THI (Eq. 3), oxy- and deoxyhemoglobin have an absorbance ratio of $\frac{{A}_{Oxy/Deoxyhemoglobin} [785 nm \dots 825 nm]}{{A}_{Oxy/Deoxyhemoglobin} [530 nm \dots 590 nm]} \sim 0.016$^33^. The corresponding absorbance ratio of melanin is $\frac{{A}_{Eumelanin} [785 nm \dots 825 nm]}{{A}_{Eumelanin} [530 nm \dots 590 nm]} \sim 0.3$^34,36^. Therefore, with increasing melanin content, the absorbance ratio $\frac{A [785 nm \dots 825 nm]}{A [530 nm \dots 590 nm]}$ increases leading to higher values of the THI.

For the StO_2_, dropping values are observed for decreasing ITA or at higher F-Class at pigmented body sites, respectively. In the calculation, second derivatives of the absorbance are used instead of the absorbance spectra, as seen in Eq. (4)^9^. Within the wavelength range of the nominator and the first addend of the denominator, the characteristic maximum of oxygenated hemoglobin is located at 757 nm, leading to a large negative value of the second derivative. Melanin has a monotone exponential decreasing absorption, which means the second derivative is positive. Combining oxygenated hemoglobin with increasing melanin content would increase the second derivative at around 577 nm. The denominator also includes in its second addend the wavelength range of λ = (740–780) nm. For oxyhemoglobin, a turning point is located in this area, associated with a second derivative of 0. Overall, both the nominator and denominator of the StO_2_ would rise with increasing melanin, but the rise in the denominator would be higher due to the sum of two wavelength ranges. This leads to decreased StO_2_ values with increasing skin pigmentation (decreasing ITA), even though this decrease is less prominent than for the NIR-index or TWI.

#### Clinical impact of the melanin influence

From the clinical perspective, it can be dangerous to miss out changes in flap perfusion occurring after surgery. In most cases, it takes at least four days until neovascularization happens between the flap and the recipient bed, so the pedicle vessel stays attached to ensure a sufficient blood flow^1^. Using the HSI-technology, it should be possible to detect a change in the tissue blood flow, e.g. directly after surgery and at several times afterwards. However, for more pigmented skin, it cannot be ensured that a malfunction of the pedicle vessel would become apparent since the HSI-evaluation procedure does not consider the influence of melanin content. In this regard, people with pigmented skin might be at higher risk for undetected tissue hypoxemia. In case the neovascularization was successful, a vascular channel should have developed at around day 7 after surgery, so the vascular pedicle can be cut between week 1 and 3 after surgery^1^. In case of unsuccessful neovascularization, the visualization of this malfunction might also be affected by the pigmentation and undetected so the flap might get necrotic. Flap failure after day 7 is quite rare^1,68^. Still, it has been shown that the most common reason for late flap failure are vascular problems, which were not detectable in Doppler examination^68^. Although this problem might be avoided when using HSI-measurements in a fairer-skinned population, people with pigmented skin are still at risk of unrecognized malperfusion.

#### Alternatives and perspectives for melanin assessment

The problem of melanin influencing perfusion measurement has also been shown in studies in which the degree of hypoxemia was incorrectly estimated due to incorrect SpO_2_ measurements^60–62^. Other studies have used optical methods to differentiate between Hb and melanin effects by comparing chromophore content in nevi and melanomas with unsuspected skin, but the majority of participants belonged to fair skin types^45,64,66^. A study on the possible use of different color spaces for heart rate measurement with video PPG showed that HSV is the most suitable^18,69^. It was possible to determine the pulse rate correctly, but a large part of the raw data was influenced by motion or illumination artefacts^69^. Currently, research is going on to differentiate Hb and melanin effects using HSI-systems on different skin types or using simulated and calculated models regarding light-tissue interaction^45,64,70^. Even though these in-vivo based models show promising results, they cannot fully depict the complexity of human skin^35,67^.

It could be promising to test if using different spectral wavelength ranges would be more robust concerning the influence of melanin or if a combination of several applications and approaches would overcome this obstacle. For example, a study using rPPG for intra- and post-operative flap perfusion monitoring used an unaffected skin area as a reference to compare the perfusion development of the newly implemented flap^16^. In an additional study, an algorithm for heart rate measurement using RGB information from rPPG was developed, which was robust to the skin tone^18,19^. These approaches could be transferred to HSI or combined with HSI-analysis to increase the quality of the calculated tissue indices. Thus, there is still a need for non-invasive and contact-free perfusion analysis methods regarding pigmentation. This might be achieved by signalling the device operator that the perfusion measurements cannot be plausible at a certain degree of pigmentation by, e.g. implementing ITA measurements and an ITA-based cut-off value. The ITA may also be used to assess the melanin concentration in the skin, as was done in another study^35^. In this, a relationship between melanin concentration and ITA was established based on a simplified model, whereby, for example, an ITA of 30° corresponded to a melanin concentration of 30 × 10^–7^ mmol/dl (± 50%). Estimated melanin concentrations can be helpful for data interpretation and establishment of future more accurate models, as melanin highly influences all of the tissue indices, especially the THI- and NIR-index. At the moment, data interpretation must be performed with caution, especially for persons with a higher degree of pigmentation.

### Age derived bias

Atherosclerosis of the flap pedicle vessel, in particular, can negatively influence the flap outcome. A study concluded that age-related arteriosclerotic changes were significant for the peroneal and radial arteries^71^. Other histopathological vessel changes were not significantly related to age but to different body sites^60^. In addition, it has been stated that flap complication rates increase with age^72^. This may be due to impaired healing after flap surgery or an impaired suitability of flaps in elder people. Regarding the second potential explanation, the influence of age on the tissue indices at multiple body sites was further investigated.

For many pigmented body sites with an ITA ≥ 55°, age showed a positive correlation with THI and a negative correlation with ITA. The THI correlated negatively with ITA.

When considering the physiology of skin aging, intrinsic and extrinsic mechanisms have to be distinguished. Intrinsic ageing is especially dependent on the decay of the levels of sexual hormones throughout the years, resulting in epidermal atrophy and less elasticity^73^. Extrinsic aging mainly depends on UV light exposure, causing tissue damage on a molecular level, resulting in, e.g. collagen loss^73^. In addition, the blood vessel size in sun-exposed skin decreases^73^, and the balance between vasodilatation and vasoconstriction of the vessels within the skin shifts on behalf of vasoconstriction^74^. A negative correlation between age and THI would be expected in this regard.

Melanin production decreases with age, and tanning capacities decrease at body sites without general sun exposure^75^. In contrast, in body sites with high general sun exposure, the pigmentation pattern of skin becomes irregular, and photo-aged skin appears to have more dopa-positive melanocytes, resulting in increased local pigmentation^75^. Furthermore, people classified as Fitzpatrick Type III and IV appear to have irreversible hyperpigmentation all over the chronically exposed area, whereas Type I and II do not^73^. These skin pigmentation alterations might explain the observed negative correlation of age with ITA, indicating more pigmentation at sun-exposed body sites with increasing age. This ITA trend might induce a bias for the THI values, causing Hb content to be overestimated with increasing age, which would be consistent with our observations. In this regard, it would be interesting to know if there is a difference between elderly participants who had chronic sun exposure throughout their lives, e.g. by working outdoors and those who tended to spend more time indoors.

At the heels and soles of the feet, age showed a negative correlation with THI and a positive correlation with ITA. The negative correlation between ITA and THI was already reported in "Melanin derived bias". The correlation of THI and ITA with age might be explained by the more frequent occurrence of hyperkeratosis at the heels of some older participants. Since keratosis appears white, the ITA values are higher and corresponding THI values are lower. In that regard, the StO_2_ measurements at the heels might also be distorted due to hyperkeratosis.

TWI correlates positively with age at different body sites. The amount of blood and therefore, fluid within the skin depends on the blood flow within the vessel systems. As stated before, the vessels are more constricted in elder people^74^, so a contrary finding would be expected. A reason why TWI correlates positively with age might lie in the data analysis procedure of the HSI-device software and the physiological fat deposition regardless of age. The absorption coefficient of water at λ = 970 nm lies close to the one of fat at λ = 930 nm^9,50,51^. In addition, when looking at the distribution of subcutaneous fat, one of the main regions where subcutaneous fat can be found is around the waist^76^. Combining this information, it can be assumed that the subcutaneous fat content within the skin of the lumbar back region, which is close to the waist, causes a bias for the TWI values. The reason for the other significant correlations between age and tissue indices listed in Table 2 are yet unclear.

### BMI derived bias

BMI has also been stated to be associated with a higher risk for venous thromboembolism after tissue transfer in head and neck surgery^77^. In addition, a higher BMI is associated with type 2 diabetes^78^, which can lead to vascular diseases such as atherosclerosis^79,80^, and so affect the tissue indices.

THI correlates positively with BMI at the palms, while ITA correlates negatively. Obesity correlates with heat production, while subcutaneous fat tissue prevents the heat from dissipating. Therefore, other mechanisms are necessary for heat release^81^. Within the dermis, arteriovenous anastomoses are located, which are responsible for thermoregulation by increasing dermal perfusion and so spreading excess heat^81^. Interestingly, these vessels can be found in the dermis of the palms and the feet but not in the forearms or calves^81,82^. This indicates that BMI-related elevated THI values might be due to increased perfusion resulting in heat loss. Furthermore, the Hb amount in the blood is higher in overweight subjects compared to their non-obese counterparts^83^.

The positive correlation between the NIR-index at the soles of the feet might be caused by thermoregulation as well since vessels open in warmer body parts^84^, e.g. after more pressure on the soles of the feet in overweight people. The same explanation might be valid for the positive correlation between BMI and StO_2_ at the soles of the feet. Still, it is yet unclear why not each of the THI, StO_2_ and NIR-index values correlated positively at both the palms and soles.

TWI correlates positively with BMI at the lumbar back regions. Most subcutaneous fat can be found, e.g. around the waist^76^ or close to the lumbar back area. As BMI correlates with the amount of subcutaneous body fat and capillary density as well as water content in the skin decrease with obesity^85^ Although capillary perfusion and water content drop, calculated TWI might be rising to the previously explained possibility that the lipid absorption at the used wavelengths gets dominant and overpowers the physiological changes with higher BMI.

Further reasons for other significant correlations between BMI and the tissue indices are still unclear.

### Sex derived bias

Another factor that possibly influences the flap's outcome is the patient’s sex. There is different information on the question, whether the female sex is a risk factor for flap failure^68^. In addition, there are sex-related differences in body composition^76,85,86^, which might influence the tissue indices.

The reduced StO_2_ in females compared to males in regions like the lumbar back region, the forearms and the right palm might be caused by the different proportions of fat and muscle as well as the thicker subcutaneous adipose tissue compared to males^85,86^. Especially premenopausal women have a deposition for subcutaneous fat accumulation, while men have more visceral fat depots^76^. As subcutaneous fat can be found mainly around the waist, the subscapular area and in the gluteal and thigh area^76^, fewer capillaries may be detected at the lumbar back region in women by the acquisition of the tissue indices since there is more fat coverage. In addition, women seem to have more fat in the extremities and lower lean mass than men^86^, which might explain the results for the palms and forearms.

The NIR-index in the palms and forearms seemed lower in women than men. Generally, women seem to have cooler fingers than men^87^ and have less perfused fingers during the cold season^88^. These findings are supported by the research of Cooke et al.^89^, who concluded that women generally have less prefunded fingers, hands and skin than men. This results from a reduced blood flood in the extremities caused by sex-related differences regarding the sympathetic tone^89^, which is a reasonable explanation for the different NIR-index findings between the two sexes.

The reason why the StO_2_ and NIR-index mean values of the two sexes are inversed at the right heel remains unclear, as well as the results for the TWI values.

### Smoking derived bias

Regarding the other presented influencing factors, it is surprising that smoking did not show any significant influence on the measurements and so the tissue indices, even though smoking has many different influences on the cardiovascular system. For example, tobacco abuse is often correlated with an increased Hb value since smoking is associated with increased levels of carbon monoxide, which binds keenly to Hb, blocking its binding sites for oxygen. The body adapts to this situation by increasing the red blood cell masses and increasing the hematocrit. Another association exists between smoking and type 2 diabetes, increasing the risk of vascular diseases^63,79^.

### The importance of the findings for future research

Even though the influencing factors discussed in the preceding sections have an impact on the results, it should be kept in mind that the correlations between body characteristics, ITA and tissue indices in this study are moderate and partly weak. This suggests that after excluding melanin as a cause of bias, the tissue indices obtained with this HSI-device are quite robust, allowing the HSI-camera to be used on different patient body parts. The results also mean that different physiological conditions can explain minor deviations when measuring two different body parts of the same person. The device user could be given a checklist of possible influencing factors and rule them out. In order to find out how large the variations are within a person, future research will be made to compare the values of the tissue indices of different parts of a person's body. In addition, future analysis will also focus on possibilities of the estimation of the melanin concentration in the skin based on the tissue indices or the full, acquired spectra by the HSI-system. Therefore, curve fitting procedures will also be investigated that may be able to describe the melanin concentration or ITA dependency of the tissue indices.

### Limiting factors

Due to the demographic situation in Germany, the number of participants with a lower ITA was limited. Hence, investigations on general influencing factors for this subpopulation still need to be performed. In addition, repeating this study with perfusion measurements of each subject in summer and winter would be of great interest to estimate the influence of different melanin amounts within the seasons depending on the amount of UV light exposure^38,44^. Regarding pigmentation classification, the Fitzpatrick skin type classification system, although commonly used to perform skin type classification^62^, is prone to inaccuracy due to subjective interpretation of personal burning or tanning tendencies^52,90^. Furthermore, regarding its history of development for psoriasis treatment, it has been stated that the Fitzpatrick Classification system is unsuitable for pigmentation classification^52,90,91^. Other methods for skin pigmentation classification are the dermoscope^56^ and colorimeter^61^, but using these devices beforehand to decide if HSI can be used for perfusion assessment is complicated for clinical implementation.

In comparison, the ITA classification is a more objective classification system since it is merely based on color and illumination parameters^53,54^. Additionally, it is suitable for skin color measurement in other studies^35,57^. Furthermore, the ITA can be obtained directly from the region of interest, thus describing the local pigmentation in relation to biophotonic measurements and related interpretations. Thus, it shows advantages over the more general Fitzpatrick classification of the whole person. In this regard, calculations of the ITA values from the RGB images could be implemented into the HSI-evaluation process to signal the operator melanin bias. The used HSI-system also has its limitations, as due to the used pushbroom technique, it is sensitive to movement artefacts, which can be corrected by immediately making a new image. The pushbroom technique does not allow a good time-resolved imaging (6 s imaging time). Therefore, other HSI-devices using snapshot technique should be used. In addition, direct surface reflections due to, e.g., water on the tissue surface or shadows during illumination will also effect the results.

## Conclusion

Free flap reconstruction is a cornerstone in treating head and neck cancer. Thus, it is even more essential to ensure proper flap perfusion over the course of time, especially during the first week after surgery. Hyperspectral Imaging offers a variety of information about the flap perfusion development both safely and quickly and is easy to apply in a clinical setting. However, interpretation must be done with caution when measurements are performed on a flap derived from a pigmented body site. Otherwise, the measured HSI-derived tissue indices may be biased, and hypoxemia of the flap could thus be missed, which may lead to flap loss. To avoid this, it could be advantageous to improve the evaluation algorithm for HSI or to implement an ITA-based cut-off value. Combining HSI with other tissue characterization techniques and evaluation methods, e.g., rPPG may also be advantageous to make the obtained results more robust against additional influences. As long as the physiological influences are kept in mind, Hyperspectral Imaging can be a valuable aid to monitor flap perfusion development, not only but especially for less pigmented people.

### Supplementary Information

Supplementary Information 1.

Supplementary Information 2.

Supplementary Information 3.

Supplementary Information 4.

Supplementary Information 5.

Supplementary Information 6.

Supplementary Information 7.

Supplementary Information 8.

## Data Availability

The raw data generated and analyzed during the study are not publicly available as data sharing was not included in the informed consent. Analysis results not included in the supplements can be obtained upon request by contacting the corresponding author.

## Abbreviations

HIS: Hyper spectral imaging
StO_2_: Tissue-oxygen-saturation
NIR: Near infrared
NIRS: Near-infrared spectroscopy
Hb: Hemoglobin
Oxy-Hb: Oxygenated hemoglobin
Deoxy-Hb: Deoxygenated hemoglobin
BMI: Body-mass-index
ITA: Individual typology angle
SpO2: Arterial oxygen saturation
UV: Ultraviolet light
THI: Tissue-hemoglobin-index
NIR-index: Near-infrared-perfusion-index
TWI: Tissue-water-index
ROI: Region of interest
F-Class: Fitzpatrick class

## References

[1] Forner D, Williams BA, Makki FM, Trites JR, Taylor SM, Hart RD (2018). Late free flap failure in head and neck reconstruction: A systematic review (in eng). Ear Nose Throat J..

[2] Bootz, F. Historical development of reconstructive surgery in head and neck oncology. *Hno. *70(6), 418–421 (2022) **(Geschichtliche****Entwicklung****der****rekonstruktiven****Chirurgie****in****der****Onkologie****des****HNO-Bereichs,****in****ger)**. 10.1007/s00106-022-01151-3 PMC9160133 35246706

[3] Nguyen JT (2013). A novel pilot study using spatial frequency domain imaging to assess oxygenation of perforator flaps during reconstructive breast surgery. Ann. Plast. Surg..

[4] Stranc MF, Sowa MG, Abdulrauf B, Mantsch HH (1998). Assessment of tissue viability using near-infrared spectroscopy. Br. J. Plast. Surg..

[5] Mücke T (2014). Changes of perfusion of microvascular free flaps in the head and neck: A prospective clinical study. Br. J. Oral Maxillofac. Surg..

[6] Kohler LH (2021). Hyperspectral Imaging (HSI) as a new diagnostic tool in free flap monitoring for soft tissue reconstruction: A proof of concept study. BMC Surg..

[7] de Weerd L, Mercer JB, Setså LB (2006). Intraoperative dynamic infrared thermography and free-flap surgery. Ann. Plast. Surg..

[8] Rao R (2009). Prediction of post-operative necrosis after mastectomy: A pilot study utilizing optical diffusion imaging spectroscopy. World J. Surg. Oncol..

[9] Holmer A, Marotz J, Wahl P, Dau M, Kämmerer PW (2018). Hyperspectral imaging in perfusion and wound diagnostics—methods and algorithms for the determination of tissue parameters. Biomed. Tech. (Berl.).

[10] Sood BG, McLaughlin K, Cortez J (2015). Near-infrared spectroscopy: Applications in neonates. Semin. Fetal Neonatal Med..

[11] Jubran A (2015). Pulse oximetry. Crit. Care.

[12] Jubran A (1999). Pulse oximetry. Crit. Care.

[13] Michaela T-W, Graham S, Gemma B, Sarah EB (2022). Noninvasive hemoglobin sensing and imaging: Optical tools for disease diagnosis. J. Biomed. Opt..

[14] Lindelauf A, Saelmans AG, van Kuijk SMJ, van der Hulst R, Schols RM (2022). Near-infrared spectroscopy (NIRS) versus hyperspectral imaging (HSI) to detect flap failure in reconstructive surgery: A systematic review. Life (Basel).

[15] Kalia S, Zhao J, Zeng H, McLean D, Kollias N, Lui H (2018). Melanin quantification by in vitro and in vivo analysis of near-infrared fluorescence. Pigment Cell Melanoma Res..

[16] Schraven SP (2023). Continuous intraoperative perfusion monitoring of free microvascular anastomosed fasciocutaneous flaps using remote photoplethysmography. Sci. Rep..

[17] Zaunseder S, Trumpp A, Wedekind D, Malberg H (2018). Cardiovascular assessment by imaging photoplethysmography—A review. Biomed. Tech. (Berl.).

[18] Kossack B, Wisotzky EL, Hilsmann A, Eisert P, Hänsch R (2019). Local blood flow analysis and visualization from RGB-video sequences. Curr. Direct. Biomed. Eng..

[19] Hammer A (2022). Camera-based assessment of cutaneous perfusion strength in a clinical setting. Physiol. Meas..

[20] Secerbegovic, A., Mesic, H., Bergsland, J., Balasingham, I. Contactless blood perfusion assessment of the free flap in breast reconstruction surgery. In *2019 13th International Symposium on Medical Information and Communication Technology (ISMICT)*, 1–4 (2019).

[21] Ruaro, B. *et al.* Laser speckle contrast analysis: Functional evaluation of microvascular damage in connective tissue diseases. Is there evidence of correlations with organ involvement, such as pulmonary damage? *Front. Physiol. 12*, 710298 (2021) **(in****eng)**. 10.3389/fphys.2021.710298 PMC8542764 34707506

[22] Tang, G. L., Kim, K. J. Laser Doppler perfusion imaging in the mouse hindlimb. *J. Vis. Exp*. 170 (2021). 10.3791/62012 33938886

[23] Kouadio AA, Jordana F, Koffi NJ, Le Bars P, Soueidan A (2018). The use of laser Doppler flowmetry to evaluate oral soft tissue blood flow in humans: A review. Arch. Oral. Biol..

[24] Stewart CJ, Frank R, Forrester KR, Tulip J, Lindsay R, Bray RC (2005). A comparison of two laser-based methods for determination of burn scar perfusion: Laser Doppler versus laser speckle imaging. Burns.

[25] Wilson SB, Spence VA (1989). Dynamic thermographic imaging method for quantifying dermal perfusion: Potential and limitations. Med. Biol. Eng. Comput..

[26] Salzhaff/Pepelow, D. V. G. S. D. A. TIVITA Tissue FAQs, Dokument: 0101001-MD-011 TIVITA Tissue FAQ_DE, Revision: C (DC-18-041) (2018).

[27] Li Q, He X, Wang Y, Liu H, Xu D, Guo F (2013). Review of spectral imaging technology in biomedical engineering: Achievements and challenges. J. Biomed. Opt..

[28] Govender, M., Chetty, K., Bulcock, H. A review of hyperspectral remote sensing and its application in vegetation and water resource studies. *Water SA. 33*(2) (2009) **(in****eng)**.

[29] Bae WK (2015). The methyltransferase EZH2 is not required for mammary cancer development, although high EZH2 and low H3K27me3 correlate with poor prognosis of ER-positive breast cancers. Mol. Carcinog..

[30] Thiem, D. G. E., Römer, P., Blatt, S., Al-Nawas, B., Kämmerer, P. W. New approach to the old challenge of free flap monitoring-hyperspectral imaging outperforms clinical assessment by earlier detection of perfusion failure. *J. Pers. Med.* **11**(11) (2021) **(in****eng)**. 10.3390/jpm11111101 PMC8625540 34834453

[31] Thoenissen, P., Heselich, A., Al-Maawi, S., Sader, R., Ghanaati, S. Hyperspectral imaging allows evaluation of free flaps in craniomaxillofacial reconstruction. *J. Craniofac. Surg. *(2022) **(in****eng)**. 10.1097/SCS.0000000000009009 36168125

[32] Guolan L, Baowei F (2014). Medical hyperspectral imaging: A review. J. Biomed. Opt..

[33] Prahl, S. *Optical Absorption of Hemoglobin*. (1999). http://omlc.ogi.edu/spectra/hemoglobin/index.html.

[34] Donner, C., Jensen, H. W. *A Spectral BSSRDF for Shading Human Skin* (The Eurographics Association, 2006).

[35] Zonios G, Bykowski J, Kollias N (2001). Skin melanin, hemoglobin, and light scattering properties can be quantitatively assessed in vivo using diffuse reflectance spectroscopy. J. Investig. Dermatol..

[36] Meglinski IV, Matcher SJ (2003). Computer simulation of the skin reflectance spectra. Comput. Methods. Progr. Biomed..

[37] Hale GM, Querry MR (1973). Optical constants of water in the 200-nm to 200-μm wavelength region. Appl. Opt..

[38] Hennessy A, Oh C, Diffey B, Wakamatsu K, Ito S, Rees J (2005). Eumelanin and pheomelanin concentrations in human epidermis before and after UVB irradiation. Pigment Cell Res..

[39] Del Bino S, Duval C, Bernerd F (2018). Clinical and biological characterization of skin pigmentation diversity and its consequences on UV impact. Int. J. Mol. Sci..

[40] Del Bino S (2015). Chemical analysis of constitutive pigmentation of human epidermis reveals constant eumelanin to pheomelanin ratio. Pigment Cell Melanoma Res..

[41] Del Bino S, Ito S, Sok J, Wakamatsu K (2022). 5,6-Dihydroxyindole eumelanin content in human skin with varying degrees of constitutive pigmentation. Pigment Cell Melanoma Res..

[42] Hunt G, Kyne S, Ito S, Wakamatsu K, Todd C, Thody A (1995). Eumelanin and phaeomelanin contents of human epidermis and cultured melanocytes. Pigment Cell Res..

[43] Thody AJ, Higgins EM, Wakamatsu K, Ito S, Burchill SA, Marks JM (1991). Pheomelanin as well as eumelanin is present in human epidermis. J. Investig. Dermatol..

[44] D'Alba L, Shawkey MD (2019). Melanosomes: Biogenesis, properties, and evolution of an ancient organelle. Physiol. Rev..

[45] Fartash V (2016). Separating melanin from hemodynamics in nevi using multimode hyperspectral dermoscopy and spatial frequency domain spectroscopy. J. Biomed. Opt..

[46] Kovatch KJ, Hanks JE, Stevens JR, Stucken CL (2019). Current practices in microvascular reconstruction in otolaryngology-head and neck surgery. Laryngoscope.

[47] Gabrysz-Forget F, Tabet P, Rahal A, Bissada E, Christopoulos A, Ayad T (2019). Free versus pedicled flaps for reconstruction of head and neck cancer defects: A systematic review. J. Otolaryngol. Head Neck Surg..

[48] McCarty JL, Corey AS, El-Deiry MW, Baddour HM, Cavazuti BM, Hudgins PA (2019). Imaging of surgical free flaps in head and neck reconstruction. AJNR Am. J. Neuroradiol..

[49] Salzhaff/Pepelow, D. V. G. S. d. A. Bedienungsanleitung Tivita Tissue: Dokument: 0101001-IM-001, Revision: H (DC-20-064) Ausgabedatum: 30.04.2020, Gültig für TIVITA Suite 1.5 (2020).

[50] Clevers JGPW, Kooistra L, Schaepman ME (2008). Using spectral information from the NIR water absorption features for the retrieval of canopy water content. Int. J. Appl. Earth Observ. Geoinf..

[51] Chung SH, Cerussi AE, Merritt SI, Ruth J, Tromberg BJ (2010). Non-invasive tissue temperature measurements based on quantitative diffuse optical spectroscopy (DOS) of water. Phys. Med. Biol..

[52] Sharma, A. N., Patel, B. C. Laser Fitzpatrick skin type recommendations. In *StatPearls* (2022). 32491558

[53] Del Bino S, Bernerd F (2013). Variations in skin colour and the biological consequences of ultraviolet radiation exposure. Br. J. Dermatol..

[54] Weatherall IL, Coombs BD (1992). Skin color measurements in terms of CIELAB color space values. J. Investig. Dermatol..

[55] Ly BCK, Dyer EB, Feig JL, Chien AL, Del Bino S (2020). Research techniques made simple: cutaneous colorimetry: A reliable technique for objective skin color measurement. J. Investig. Dermatol..

[56] Hanlon, K. L., Wei, G., Correa-Selm, L., Grichnik, J. M. Dermoscopy and skin imaging light sources: a comparison and review of spectral power distribution and color consistency. *J. Biomed. Opt. 27*(8) (2022) **(in****eng)**. 10.1117/1.JBO.27.8.080902 PMC9360608 36452032

[57] Cho C, Ruan P, Lee E, Ha J (2015). Comparison of skin color between two Asian populations: according to latitude and UV exposure. J. Cosmet. Dermatol..

[58] Osto M, Hamzavi IH, Lim HW, Kohli I (2022). Individual typology angle and Fitzpatrick skin phototypes are not equivalent in photodermatology. Photochem. Photobiol..

[59] Linek M (2022). Evaluation of hyperspectral imaging to quantify perfusion changes during the modified Allen test. Lasers Surg. Med..

[60] Holder AL, Wong AI (2022). The big consequences of small discrepancies: Why racial differences in pulse oximetry errors matter. Crit. Care Med..

[61] Fawzy A (2022). Racial and ethnic discrepancy in pulse oximetry and delayed identification of treatment eligibility among patients with COVID-19. JAMA Intern. Med..

[62] Cabanas, A. M., Fuentes-Guajardo, M., Latorre, K., Leon, D., Martin-Escudero, P. Skin pigmentation influence on pulse oximetry accuracy: A systematic review and bibliometric analysis. *Sensors (Basel)*. **22**(9), (2022). 10.3390/s22093402 PMC9102088 35591092

[63] Shah N, Osea EA, Martinez GJ (2014). Accuracy of noninvasive hemoglobin and invasive point-of-care hemoglobin testing compared with a laboratory analyzer. Int. J. Lab. Hematol..

[64] Nkengne A, Robic J, Seroul P, Gueheunneux S, Jomier M, Vie K (2018). SpectraCam(®): A new polarized hyperspectral imaging system for repeatable and reproducible in vivo skin quantification of melanin, total hemoglobin, and oxygen saturation. Skin Res. Technol..

[65] Zonios G, Dimou A, Bassukas I, Galaris D, Tsolakidis A, Kaxiras E (2008). Melanin absorption spectroscopy: New method for noninvasive skin investigation and melanoma detection. J. Biomed. Opt..

[66] Ilona K (2011). Towards noncontact skin melanoma selection by multispectral imaging analysis. J. Biomed. Opt..

[67] Saurabh V, Amit B, Philippe B (2013). Estimating physiological skin parameters from hyperspectral signatures. J. Biomed. Opt..

[68] Yu P, Chang DW, Miller MJ, Reece G, Robb GL (2009). Analysis of 49 cases of flap compromise in 1310 free flaps for head and neck reconstruction. Head Neck.

[69] Gill RT, Zheng L (2015). On the benefits of alternative color spaces for noncontact heart rate measurements using standard red-green-blue cameras. J. Biomed. Opt..

[70] Main M, Pilkington RJJ, Gibson GM, Kallepalli A (2022). Simulated assessment of light transport through ischaemic skin flaps. Br. J. Oral Maxillofac. Surg..

[71] Ghassemi A, Köhlen D, Braunschweig T, Modabber A, Prescher A, Nanhekhan L (2016). Histopathological differences of the pedicle artery in commonly used free flaps: The influence of age, gender, and side. J. Oral Maxillofac. Surg..

[72] Hwang K, Lee JP, Yoo SY, Kim H (2016). Relationships of comorbidities and old age with postoperative complications of head and neck free flaps: A review. J. Plast. Reconstr. Aesthet. Surg..

[73] Kohl E, Steinbauer J, Landthaler M, Szeimies RM (2011). Skin ageing. J. Eur. Acad. Dermatol. Venereol..

[74] Rorteau, J., Chevalier, F. P., Fromy, B., Lamartine, J. Functional integrity of aging skin, from cutaneous biology to anti-aging strategies. *Med. Sci. (Paris)*. **36**(12), 1155–1162 (2020). **(Vieillissement****et****intégrité****de****la****peau****-****De****la****biologie****cutanée****aux****stratégies****anti-âge,****in****fre)**. 10.1051/medsci/2020223 33296632

[75] Rittié, L., Fisher, G. J. Natural and sun-induced aging of human skin. *Cold Spring Harb. Perspect. Med.* **5**(1), a015370 (2015) **(in****eng)**. 10.1101/cshperspect.a015370 PMC4292080 25561721

[76] Palmer BF, Clegg DJ (2015). The sexual dimorphism of obesity. Mol. Cell Endocrinol..

[77] Saadoun R (2022). Assessment of BMI and venous thromboembolism rates in patients on standard chemoprophylaxis regimens after undergoing free tissue transfer to the head and neck. JAMA Otolaryngol. Head Neck Surg..

[78] Looker HC, Knowler WC, Hanson RL (2001). Changes in BMI and weight before and after the development of type 2 diabetes. Diabetes Care.

[79] U. S. D. of Health and H. Services, *How Tobacco Smoke Causes Disease: The Biologyand Behavioral Basis for Smoking-Attributable Disease: A Report of the Surgeon Genera* (U.S. Department of Health and Human Services, Centers for Disease Control and Prevention, National Center for Chronic Disease Prevention and Health Promotion, Office on Smoking and Health, 2010).

[80] Stratmann, B. Dicarbonyl stress in diabetic vascular disease. *Int. J. Mol. Sci*. **23**(11) (2022) **(in****eng)**. 10.3390/ijms23116186 PMC9181283 35682865

[81] Savastano DM, Gorbach AM, Eden HS, Brady SM, Reynolds JC, Yanovski JA (2009). Adiposity and human regional body temperature. Am. J. Clin. Nutr..

[82] Cracowski J-L, Roustit M (2020). Human skin microcirculation. Compr. Physiol..

[83] Akter R, Nessa A, Sarker D, Yesmin M (2017). Effect of obesity on hemoglobin concentration. Mymensingh. Med. J..

[84] Wang Y, Zhu K, Wang J, Yang L (2019). Numerical simulation of heat induced flow-mediated dilation of blood vessels. J. Therm. Biol..

[85] Rodriguez, A. J. *et al.* Skin optical properties in the obese and their relation to body mass index: A review. *J. Biomed. Opt*. **27**(3), (2022) **(in****eng)**. 10.1117/1.JBO.27.3.030902 PMC8963797 35352513

[86] Schorr M (2018). Sex differences in body composition and association with cardiometabolic risk. Biol. Sex Differ..

[87] Kim H, Richardson C, Roberts J, Gren L, Lyon JL (1998). Cold hands, warm heart. Lancet.

[88] Gardner-Medwin JM, Macdonald IA, Taylor JY, Riley PH, Powell RJ (2001). Seasonal differences in finger skin temperature and microvascular blood flow in healthy men and women are exaggerated in women with primary Raynaud's phenomenon. Br. J. Clin. Pharmacol..

[89] Cooke JP, Creager MA, Osmundson PJ, Shepherd JT (1990). Sex differences in control of cutaneous blood flow. Circulation.

[90] Eilers S (2013). Accuracy of self-report in assessing Fitzpatrick skin phototypes I through VI. JAMA Dermatol..

[91] Fitzpatrick TB (1988). The validity and practicality of sun-reactive skin types I through VI. Arch. Dermatol..

