# Supplementary material for: Investigation on the influence of the skin tone on hyperspectral imaging for free flap surgery
Source: Sci Rep. 2024 Jun 17;14:13979. doi: 10.1038/s41598-024-64549-9 (PMC11183063; doi:10.1038/s41598-024-64549-9)
Supplement: Supplementary file 2 — Supplementary Information 2. [file 41598_2024_64549_MOESM2_ESM.pdf]

Investigation on the influence of the skin tone on Hyperspectral Imaging for free flap surgery

Pachyn, Ester*; Aumiller, Maximilian; Freymüller, Christian; Linek, Matthäus; Volgger, Veronika; Buchner, Alexander; Rühm, Adrian, Sroka, Ronald

Supplement 2:

p- value results for correlation test between F-Class and ITA at different body sites.

	neck	lumbar back right	lumbar back left	dorsum right hand
V/VI vs. IV	0.010	0.006	0.038	< 0.001
V/VI vs. III	< 0.001	< 0.001	< 0.001	< 0.001
V/VI vs. II	< 0.001	< 0.001	< 0.001	< 0.001
V/VI vs. I	< 0.001	< 0.001	< 0.001	< 0.001
IV vs. III	0.478	1	0.760	1
IV vs. II	0.473	0.006	0.006	1
IV vs. I	0.013	0.015	0.001	0.059
III vs. II	1	0.436	1	1
III vs. I	0.659	0.284	0.056	0.437
II vs. I	0.664	1	1	0.508

	dorsum left hand	volar right forearm	volar left forearm	dorsum of foot right
V/VI vs. IV	0.001	0.007	0.003	0.047
V/VI vs. III	< 0.001	< 0.001	< 0.001	< 0.001
V/VI vs. II	< 0.001	< 0.001	< 0.001	< 0.001
V/VI vs. I	< 0.001	< 0.001	< 0.001	< 0.001
IV vs. III	00.53	0.543	0.414	0.163
IV vs. II	1	0.244	0.610	0.077
IV vs. I	0.029	0.018	0.007	0.009
III vs. II	1	1	1	1
III vs. I	0.368	0.738	0.495	0.973
II vs. I	1	1	0.376	1

	dorsum of foot left	abdomen	palm right hand	palm left hand
V/VI vs. IV	0.173	0.036	0.019	0.049
V/VI vs. III	< 0.001	< 0.001	< 0.001	< 0.001
V/VI vs. II	< 0.001	< 0.001	< 0.001	< 0.001
V/VI vs. I	< 0.001	< 0.001	< 0.001	< 0.001
IV vs. III	0.044	0.565	1	0.224
IV vs. II	0.004	< 0.001	1	0.823
IV vs. I	0.014	0.002	0.317	0.041
III vs. II	1	0.185	1	1
III vs. I	1	0.140	1	1
II vs. I	1	1	1	0.965

	heel right	heel left	sole of right foot	sole of left foot
V/VI vs. IV	1	1	1	1
V/VI vs. III	1	1	0.021	0.003
V/VI vs. II	0.056	0.950	0.004	< 0.001
V/VI vs. I	0.520	1	0.672	0.208
IV vs. III	0.984	0.893	00.044	0.117
IV vs. II	0.017	0.115	0.008	0.018
IV vs. I	0.418	1	1	1
III vs. II	1	1	1	1
III vs. I	0.015	0.213	1	1
II vs. I	< 0.001	0.041	1	1
