# Supplementary material for: Investigation on the influence of the skin tone on hyperspectral imaging for free flap surgery
Source: Sci Rep. 2024 Jun 17;14:13979. doi: 10.1038/s41598-024-64549-9 (PMC11183063; doi:10.1038/s41598-024-64549-9)
Supplement: Supplementary file 3 — Supplementary Information 3. [file 41598_2024_64549_MOESM3_ESM.pdf]

Investigation on the influence of the skin tone on Hyperspectral Imaging for free flap surgery

Pachyn, Ester*; Aumiller, Maximilian; Freymüller, Christian; Linek, Matthäus; Volgger, Veronika; Buchner, Alexander; Rühm, Adrian, Sroka, Ronald

Supplement 3:

Significant differences of the tissue indices between the F-Classes at different body sites

body site	tissue indices	F-Classes	p-value
neck (n=101)	NIR-index	V vs. IV	0.025
		V vs. III	< 0.001
		V vs. II	< 0.001
		V vs. I	< 0.001
		VI vs. II	0.008
		VI vs. I	<0.001
	StO2	V vs. IV	0.015
		V vs. III	< 0.001
		V vs. II	< 0.001
		V vs. I	< 0.001
		IV vs. III	0.043
		IV vs. II	0.033
		IV vs. I	0.048
	THI	V vs. IV	0.004
		V vs. III	< 0.001
		V vs. II	< 0.001
		V vs. I	<0.001
		IV vs. I	0.005
	TWI	V vs. IV	0.014
		V vs. III	< 0.001
		V vs. II	< 0.001
		V vs. I	< 0.001
		IV vs. II	0.024
lumbar back right (n=100)	NIR-index	V vs. III	< 0.001
		V vs. II	< 0.001
		V vs. I	< 0.001
		IV vs. II	< 0.001
		IV vs. I	< 0.001
		III vs. I	0.013
	StO2	V vs. IV	0.037
		V vs. III	< 0.001
		V vs. II	< 0.001
		V vs. I	< 0.001
		IV vs. II	0.001
		IV vs. I	0.019
	THI	V vs. IV	0.039
		V vs. III	< 0.001
		V vs. II	< 0.001
		V vs. I	< 0.001
		IV vs. II	< 0.001

		IV vs. I	0.001
	TWI	V vs. IV	0.003
		V vs. III	< 0.001
		V vs. II	< 0.001
		V vs. I	< 0.001
lumbar back left (n=100)	NIR-index	V vs. III	< 0.001
		V vs. II	< 0.001
		V vs. I	< 0.001
		IV vs. III	0.024
		IV vs. II	< 0.001
		IV vs. I	< 0.001
	StO2	V vs. IV	0.022
		V vs. III	< 0.001
		V vs. II	< 0.001
		V vs. I	< 0.001
		IV vs. II	0.003
		IV vs. I	0.024
	THI	V vs. IV	0.035
		V vs. III	< 0.001
		V vs. II	< 0.001
		V vs. I	< 0.001
		IV vs. II	0.001
		IV vs. I	< 0.001
		III vs. I	0.045
	TWI	V vs. IV	0.012
		V vs. III	< 0.001
		V vs. II	< 0.001
		V vs. I	0.003
		IV vs. III	0.018
		IV vs. II	0.047
dorsum of hand right (n=101)	NIR-index	V vs. IV	0.012
		V vs. III	< 0.001
		V vs. II	< 0.001
		V vs. I	< 0.001
		VI vs. II	0.012
		VI vs. I	0.001
	StO2	V vs. IV	0.003
		V vs. III	< 0.001
		V vs. II	< 0.001
		V vs. I	< 0.001
		IV vs. II	0.05
	THI	V vs. IV	0.001
		V vs. III	< 0.001
		V vs. II	< 0.001
		V vs. I	< 0.001
		IV vs. I	0.016
	TWI	V vs. IV	0.004
		V vs. III	< 0.001
		V vs. II	< 0.001
		V vs. I	< 0.001
		IV vs. I	0.029
dorsum of hand left (n=101)	NIR-index	V vs. IV	0.014
		V vs. III	< 0.001

		V vs. II	< 0.001
		V vs. I	< 0.001
		VI vs. II	0.003
		VI vs. I	0.001
	StO2	V vs. IV	0.003
		V vs. III	< 0.001
		V vs. II	< 0.001
		V vs. I	< 0.001
		IV vs. II	0.026
	THI	V vs. IV	0.001
		V vs. III	<0.001
		V vs. II	< 0.001
		V vs. I	< 0.001
	TWI	V vs. IV	0.003
		V vs. III	< 0.001
		V vs. II	< 0.001
		V vs. I	< 0.001
		IV vs. I	0.018
forearm right (n=101)	NIR-index	V vs. IV	0.025
		V vs. III	< 0.001
		V vs. II	< 0.001
		V vs. I	< 0.001
		IV vs. II	0.002
		IV vs. I	0.036
	StO2	V vs. III	< 0.001
		V vs. II	< 0.001
		V vs. I	0.009
		IV vs. II	0.022
	THI	V vs. IV	0.004
		V vs. III	< 0.001
		V vs. II	< 0.001
		V vs. I	< 0.001
		IV vs. I	0.033
	TWI	V vs. III	< 0.001
		V vs. II	< 0.001
		V vs. I	< 0.001
		IV vs. I	0.006
forearm left (n=101)	NIR-index	V vs. IV	0.006
		V vs. III	< 0.001
		V vs. II	< 0.001
		V vs. I	< 0.001
		IV vs. II	0.037
		IV vs. I	0.002
	StO2	V vs. IV	0.009
		V vs. III	< 0.001
		V vs. II	< 0.001
		V vs. I	0.001
	THI	V vs. IV	0.005
		V vs. III	< 0.001
		V vs. II	< 0.001
		V vs. I	< 0.001
		IV vs. I	0.001
	TWI	V vs. IV	0.013

		V vs. III	< 0.001
		V vs. II	0.001
		V vs. I	< 0.001
		IV vs. I	0.028
dorsum of foot right (n=99)	NIR-index	V vs. IV	0.05
		V vs. III	< 0.001
		V vs. II	< 0.001
		V vs. I	< 0.001
		VI vs. II	< 0.001
		VI vs. I	0.001
	StO2	V vs. IV	0.001
		V vs. III	< 0.001
		V vs. II	< 0.001
		V vs. I	< 0.001
	THI	V vs. III	< 0.001
		V vs. II	< 0.001
		V vs. I	<0.001
		IV vs. III	0.025
		IV vs. II	0.001
		IV vs. I	0.005
	TWI	V vs. IV	0.001
		V vs. III	< 0.001
		V vs. II	< 0.001
		V vs. I	< 0.001
		IV vs. I	0.043
dorsum of foot left (n=100)	NIR-index	V vs. III	< 0.001
		V vs. II	< 0.001
		V vs. I	< 0.001
		VI vs. II	< 0.001
		VI vs. I	< 0.001
	StO2	V vs. IV	0.02
		V vs. III	< 0.001
		V vs. II	< 0.001
		V vs. I	< 0.001
	THI	V vs. IV	0.048
		V vs. III	< 0.001
		V vs. II	< 0.001
		V vs. I	< 0.001
		IV vs. II	0.002
		IV vs. I	0.004
	TWI	V vs. IV	0.001
		V vs. III	< 0.001
		V vs. II	< 0.001
		V vs. I	< 0.001
		IV vs. I	0.042
abdomen (n=101)	NIR-index	V vs. IV	0.027
		V vs. III	< 0.001
		V vs. II	< 0.001
		V vs. I	< 0.001
		VI vs. II	0.001
		VI vs. I	< 0.001
	StO2	V vs. IV	0.029
		V vs. III	< 0.001

	V vs. II	< 0.001
	V vs. I	< 0.001
THI	V vs. IV	0.041
	V vs. III	< 0.001
	V vs. II	< 0.001
	V vs. I	< 0.001
	IV vs. II	< 0.001
	IV vs. I	< 0.001
TWI	V vs. IV	0.02
	V vs. III	< 0.001
	V vs. II	< 0.001
	V vs. I	< 0.001
