# Supplementary material for: Investigation on the influence of the skin tone on hyperspectral imaging for free flap surgery
Source: Sci Rep. 2024 Jun 17;14:13979. doi: 10.1038/s41598-024-64549-9 (PMC11183063; doi:10.1038/s41598-024-64549-9)
Supplement: Supplementary file 4 — Supplementary Information 4. [file 41598_2024_64549_MOESM4_ESM.pdf]

Investigation on the influence of the skin tone on Hyperspectral Imaging for free flap surgery

Pachyn, Ester*; Aumiller, Maximilian; Freymüller, Christian; Linek, Matthäus; Volgger, Veronika; Buchner, Alexander; Rühm, Adrian, Sroka, Ronald

Supplement 4:

Mean and standard deviation of the tissue indices, pigmented body sites

body site	Class	NIR-index (a.u.)	StO2 (%)	THI (a.u.)	TWI (a.u.)
neck	I	53 ± 6	69 ± 8	13 ± 13	41 ± 3
	II	44 ± 12	66 ± 13	31 ± 21	41 ± 6
	III	41 ± 10	65 ± 12	33 ± 17	40 ± 5
	IV	28 ± 15	53 ± 12	41 ± 17	36 ± 5
	V/VI	3 ± 8	32 ± 10	83 ± 19	22 ± 11

body site	Class	NIR-index (a.u.)	StO2 (%)	THI (a.u.)	TWI (a.u.)
lumbar back right	I	56 ± 4	57 ± 11	8 ± 8	54 ± 4
	II	49 ± 6	54 ± 11	12 ± 11	54 ± 6
	III	43 ± 9	50 ± 13	21 ± 11	54 ± 6
	IV	28 ± 15	40 ± 9	36 ± 19	50 ± 6
	V/VI	2 ± 6	22 ± 10	83 ± 17	27 ± 15

body site	F-Class	NIR-index (a.u.)	StO2 (%)	THI (a.u.)	TWI (a.u.)
lumbar back left	I	54 ± 4	58 ± 12	5 ± 6	52 ± 4
	II	49 ± 6	55 ± 11	13 ± 11	54 ± 6
	III	44 ± 10	50 ± 12	20 ± 11	55 ± 6
	IV	28 ± 15	42 ± 9	34 ± 18	48 ± 6
	V/VI	1 ± 5	24 ± 10	86 ± 19	26 ± 15

body site	F-Class	NIR-index (a.u.)	StO2 (%)	THI (a.u.)	TWI (a.u.)
dorsum of hand right	I	52 ± 5	60 ± 9	16 ± 10	44 ± 4
	II	47 ± 9	60 ± 9	31 ± 13	40 ± 5
	III	45 ± 7	57 ± 9	29 ± 11	42 ± 6
	IV	35 ± 12	51 ± 10	36 ± 15	37 ± 5
	V/VI	4 ± 9	33 ± 8	78 ± 18	25 ± 9

body site	F-Class	NIR-index (a.u.)	StO2 (%)	THI (a.u.)	TWI (a.u.)
dorsum of hand left	I	54 ± 5	61 ± 9	19 ± 13	44 ± 3
	II	49 ± 10	61 ± 10	27 ± 11	40 ± 6
	III	45 ± 9	55 ± 8	29 ± 13	39 ± 6
	IV	36 ± 11	52 ± 9	36 ± 14	38 ± 4
	V/VI	5 ± 10	34 ± 8	77 ± 18	25 ± 10

body site	F-Class	NIR-index (a.u.)	StO2 (%)	THI (a.u.)	TWI (a.u.)
forearm right	I	51 ± 6	51 ± 10	11 ± 12	44 ± 3
	II	51 ± 8	54 ± 9	17 ± 11	40 ± 5
	III	48 ± 7	52 ± 8	18 ± 9	40 ± 5
	IV	39 ± 10	46 ± 8	25 ± 12	38 ± 5
	V/VI	7 ± 13	36 ± 8	67 ± 22	28 ± 10

body site	F-Class	NIR-index (a.u.)	StO2 (%)	THI (a.u.)	TWI (a.u.)
forearm left	I	55 ± 5	52 ± 10	7 ± 6	45 ± 3
	II	50 ± 8	53 ± 9	16 ± 11	40 ± 6
	III	48 ± 7	50 ± 8	18 ± 8	41 ± 5
	IV	41 ± 10	47 ± 8	24 ± 9	39 ± 5
	V/VI	8 ± 13	36 ± 8	66 ± 21	30 ± 10

body site	F-Class	NIR-index (a.u.)	StO2 (%)	THI (a.u.)	TWI (a.u.)
dorsum of foot right	I	47 ± 6.11	46 ± 4	18 ± 11	46 ± 3
	II	45± 6.20	44 ± 6	21 ± 9	41 ± 6
	III	40 ± 7.02	42 ± 6	24 ± 8	41 ± 6
	IV	28 ± 14	39 ± 8	38 ± 14	40 ± 6
	V/VI	3 ± 8	24 ± 6	78 ± 18	25 ± 10

body site	F-Class	NIR-index (a.u.)	StO2 (%)	THI (a.u.)	TWI (a.u.)
dorsum of foot left	I	48 ± 7	46 ± 4	15 ± 13	47± 4
	II	46 ± 7	45 ± 8	21 ±9	42± 6
	III	40 ± 9	42 ± 7	25± 11	41± 6
	IV	28 ± 11	40 ± 8	37± 13	40 ± 6
	V/VI	3 ± 9	24 ± 6	79± 19	24 ± 10

body site	F-Class	NIR-index (a.u.)	StO2 (%)	THI (a.u.)	TWI (a.u.)
abdomen	I	54 ± 5	48 ± 6	3 ± 5	49 ± 3
	II	49 ± 7	45 ± 8	5 ± 5	47 ± 5
	III	44 ± 9	44 ± 9	12 ± 10	48 ± 7
	IV	34 ± 13	38 ± 9	22 ± 16	45 ± 5
	V/VI	3 ± 8	26 ± 10	70 ± 20	28 ± 12
