# Supplementary material for: Investigation on the influence of the skin tone on hyperspectral imaging for free flap surgery
Source: Sci Rep. 2024 Jun 17;14:13979. doi: 10.1038/s41598-024-64549-9 (PMC11183063; doi:10.1038/s41598-024-64549-9)
Supplement: Supplementary file 5 — Supplementary Information 5. [file 41598_2024_64549_MOESM5_ESM.pdf]

Investigation on the influence of the skin tone on Hyperspectral Imaging for free flap surgery

Pachyn, Ester*; Aumiller, Maximilian; Freymüller, Christian; Linek, Matthäus; Volgger, Veronika; Buchner, Alexander; Rühm, Adrian, Sroka, Ronald

Supplement 5:

Significant differences of the tissue indices between the F-Classes, less pigmented body sites

body site	tissue indices	F-Classes	p-value
palm right (n=101)	NIR-index	V vs. IV	0.003
		V vs. III	< 0.001
		V vs. II	< 0.001
		V vs. I	0.006
	THI	V vs. IV	0.021
		V vs. III	< 0.001
		V vs. II	< 0.001
		V vs. I	< 0.001
palm left (n=101)	NIR-index	V vs. IV	0.002
		V vs. III	< 0.001
		V vs. II	< 0.001
		V vs. I	< 0.001
		IV vs. II	0.018
	THI	V vs. IV	0.022
		V vs. III	< 0.001
		V vs. II	< 0.001
		V vs. I	< 0.001
		IV vs. I	0.011
	TWI	V vs. IV	0.01
		V vs. III	0.004
		V vs. II	0.002
		V vs. I	0.019
heel right (n=100)	NIR-index	V vs. I	0.026
	THI	V vs. III	0.007
		V vs. II	< 0.001
		IV vs. II	0.017
		II vs. I	0.011
heel left (n=100)	THI	V vs. III	0.041
		V vs. II	0.001
		IV vs. II	0.045
		II vs. I	0.03
sole of the foot right (n=99)	THI	V vs. III	0.004
		V vs. II	0.001
		IV vs. II	0.023
sole of the foot left (n=100)	THI	V vs. III	0.004
		V vs. II	< 0.001
