# Supplementary material for: Investigation on the influence of the skin tone on hyperspectral imaging for free flap surgery
Source: Sci Rep. 2024 Jun 17;14:13979. doi: 10.1038/s41598-024-64549-9 (PMC11183063; doi:10.1038/s41598-024-64549-9)
Supplement: Supplementary file 6 — Supplementary Information 6. [file 41598_2024_64549_MOESM6_ESM.pdf]

Investigation on the influence of the skin tone on Hyperspectral Imaging for free flap surgery

Pachyn, Ester*; Aumiller, Maximilian; Freymüller, Christian; Linek, Matthäus; Volgger, Veronika; Buchner, Alexander; Rühm, Adrian, Sroka, Ronald

Supplement 6:

Mean and standard deviation of the tissue indices, less pigmented body sites

body site	F-Class	NIR-index (a.u.)	StO2 (%)	THI (a.u.)	TWI (a.u.)
heel right	I	67 ± 6	77 ± 11	45 ± 11	56 ± 5
	II	61 ± 7	68 ± 11	27 ± 13	55 ± 8
	III	60 ± 8	68 ± 14	32 ± 12	54 ± 8
	IV	62 ± 7	74 ± 8	41 ± 14	54 ± 6
	V/VI	57 ± 9	74 ± 7	46 ± 13	56 ± 6

body site	F-Class	NIR-index (a.u.)	StO2 (%)	THI (a.u.)	TWI (a.u.)
heel left	I	64 ± 6	73 ± 13	44 ± 10	58 ± 6
	II	59 ± 7	66 ± 12	28 ± 12	54 ± 9
	III	60 ± 8	67 ± 12	33 ± 12	54 ± 8
	IV	62 ± 8	73 ± 10	40 ± 13	53 ± 7
	V/VI	57 ± 8	73 ± 7	45 ± 11	55 ± 6

body site	F-Class	NIR-index (a.u.)	StO2 (%)	THI (a.u.)	TWI (a.u.)
palm right	I	55 ± 9	61 ± 14	30 ± 14	51 ± 3
	II	57 ± 7	69 ± 9	33 ± 12	48 ± 6
	III	56 ± 7	67 ± 8	31 ± 10	49 ± 6
	IV	52 ± 6	67 ± 8	39 ± 9	47 ± 6
	V/VI	36 ± 14	64 ± 10	55 ± 11	45 ± 5

body site	F-Class	NIR-index (a.u.)	StO2 (%)	THI (a.u.)	TWI (a.u.)
palm left	I	56 ± 6	67 ± 12	24 ± 10	49 ± 3
	II	58 ± 6	70 ± 8	31 ± 10	49 ± 6
	III	55 ± 7	66 ± 8	33 ± 10	48 ± 5
	IV	52 ± 6	68 ± 10	40 ± 10	48 ± 5
	V/VI	30 ± 12	63 ± 11	55 ± 11	42 ± 6

body site	F-Class	NIR-index (a.u.)	StO2 (%)	THI (a.u.)	TWI (a.u.)
sole right	I	55 ± 8	55 ± 13	29 ± 11	57 ± 4
	II	56 ± 6	60 ± 10	24 ± 10	58 ± 6
	III	56 ± 5	61 ± 11	25 ± 11	58 ± 6
	IV	53 ± 7	57 ± 10	34 ± 12	58 ± 6
	V/VI	52 ± 9	59 ± 9	39 ± 12	59 ± 6

body site	F-Class	NIR-index (a.u.)	StO2 (%)	THI (a.u.)	TWI (a.u.)
sole left	I	54 ± 9	56 ± 12	27 ± 12	55 ± 4
	II	56 ± 6	60 ± 11	25 ± 11	58 ± 6
	III	55 ± 5	61 ± 10	26 ± 11	57 ± 7
	IV	51 ± 9	59 ± 11	32 ± 13	55 ± 6
	V/VI	53 ± 9	63 ± 9	39 ± 8	57 ± 5
