# Supplementary material for: Investigation on the influence of the skin tone on hyperspectral imaging for free flap surgery
Source: Sci Rep. 2024 Jun 17;14:13979. doi: 10.1038/s41598-024-64549-9 (PMC11183063; doi:10.1038/s41598-024-64549-9)
Supplement: Supplementary file 7 — Supplementary Information 7. [file 41598_2024_64549_MOESM7_ESM.pdf]

Investigation on the influence of the skin tone on Hyperspectral Imaging for free flap surgery

Pachyn, Ester*; Aumiller, Maximilian; Freymüller, Christian; Linek, Matthäus; Volgger, Veronika; Buchner, Alexander; Rühm, Adrian, Sroka, Ronald

Supplement 7:

Correlation between the tissue indices and the ITA at different body sites

body site	tissue indices	r-value	p-value
neck	NIR-index	0.644	<0.001
	StO2	0.319	<0.001
	THI	-0.9	<0.001
	TWI	0.506	<0.001
lumbar back right	NIR-index	0.746	<0.001
	StO2	0.431	<0.001
	THI	-0.928	<0.001
	TWI	0.416	<0.001
lumbar back left	NIR-index	0.76	<0.001
	StO2	0.435	<0.001
	THI	-0.929	<0.001
	TWI	0.456	<0.001
dorsum of hand right	NIR-index	0.692	<0.001
	StO2	0.416	<0.001
	THI	-0.875	<0.001
	TWI	0.482	<0.001
dorsum of hand left	NIR-index	0.702	<0.001
	StO2	0.484	<0.001
	THI	-0.851	<0.001
	TWI	0.469	<0.001
forearm right	NIR-index	0.694	<0.001
	StO2	0.345	<0.001
	THI	-0.878	<0.001
	TWI	0.469	<0.001
forearm left	NIR-index	0.626	<0.001
	StO2	0.283	<0.001
	THI	-0.839	<0.001
	TWI	0.525	<0.001
dorsum of foot right	NIR-index	0.660	<0.001
	StO2	0.538	<0.001
	THI	-0.806	<0.001
	TWI	0.448	<0.001
dorsum of foot left	NIR-index	0.698	<0.001
	StO2	0.399	<0.001
	THI	-0.851	<0.001
	TWI	0.518	<0.001
abdomen	NIR-index	0.814	<0.001
	StO2	0.488	<0.001
	THI	-0.93	<0.001
	TWI	0.537	<0.001

heel right	NIR-index	-0.387	<0.001
	StO2	-0.611	<0.001
	THI	-0.886	<0.001
	TWI	-0.161	0.11
heel left	NIR-index	-0.347	<0.001
	StO2	-0.416	<0.001
	THI	-0.818	<0.001
	TWI	-0.135	0.181
palm right	NIR-index	0.358	<0.001
	StO2	-0.22	0.828
	THI	-0.801	<0.001
	TWI	0.171	0.087
palm left	NIR-index	0.364	<0.001
	StO2	0.099	0.324
	THI	-0.838	<0.001
	TWI	0.174	0.082
sole right	NIR-index	-0.126	0.213
	StO2	-0.344	<0.001
	THI	-0.83	<0.001
	TWI	0.154	0.129
sole left	NIR-index	0.034	0.734
	StO2	-0.189	0.059
	THI	-0.866	<0.001
	TWI	0.158	0.117
