# Supplementary material for: Investigation on the influence of the skin tone on hyperspectral imaging for free flap surgery
Source: Sci Rep. 2024 Jun 17;14:13979. doi: 10.1038/s41598-024-64549-9 (PMC11183063; doi:10.1038/s41598-024-64549-9)
Supplement: Supplementary file 8 — Supplementary Information 8. [file 41598_2024_64549_MOESM8_ESM.pdf]

Investigation on the influence of the skin tone on Hyperspectral Imaging for free flap surgery

Pachyn, Ester*; Aumiller, Maximilian; Freymüller, Christian; Linek, Matthäus; Volgger, Veronika; Buchner, Alexander; Rühm, Adrian, Sroka, Ronald

Supplement 8:

MatLab Program for conversion of RGB values to LAB and calculation of ITA.

```
%% Extraction of RGB values

        R = mean(squeeze(double(ROI_RGB(:,:,1))),'all');
        G = mean(squeeze(double(ROI_RGB(:,:,2))),'all');
        B = mean(squeeze(double(ROI_RGB(:,:,3))),'all');

         % STD
         Rc = squeeze(double(ROI_RGB(:,:,1)));
         Gc = squeeze(double(ROI_RGB(:,:,2)));
         Bc = squeeze(double(ROI_RGB(:,:,3)));

         Rstd = std(double(Rc(:)));
         Gstd = std(double(Gc(:)));
         Bstd = std(double(Bc(:)));

         RGB = [R G B];
         RGBstd = [Rstd Gstd Bstd];

% RGB values normalized to 0-1 by division with 255 (rgb2lab function needs value
range from 0 - 1 -> see function documentation)

       LAB=rgb2lab(RGB./255,'WhitePoint','d50','ColorSpace','srgb');

% Calculation of ITA as in:
% S. Del Bino und F. Bernerd, "Variations in skin colour and the biological
consequences of ultraviolet radiation exposure", Br J Dermatol, Bd. 169, S. 33-40,
Okt. 2013, doi: 10.1111/bjd.12529.

       ITA = atan((LAB(1)-50)/LAB(3))*180/pi;
```
